# Comparative effectiveness of ultrathin vs. standard strut drug-eluting stents: insights from a large-scale meta-analysis with extended follow-up

**DOI:** 10.1186/s40001-024-01949-7

**Published:** 2024-07-27

**Authors:** Ahmed Hassan, Ahmed Mazen Amin, Ahmed Farid Gadelmawla, Ahmed Mansour, Hamed Abdelma’aboud Mostafa, Mariam Tarek Desouki, Mostafa Mahmoud Naguib, Bilal Ali, Aisha Siraj, Mustafa Suppah, Diaa Hakim

**Affiliations:** 1 Faculty of Medicine, October 6 University, Giza, Egypt; 2 Department of Cardiology, Suez Medical Complex, Ministry of Health and Population, Suez, Egypt; 3 Faculty of Medicine, Mansoura University, Mansoura, Egypt; 4 Faculty of Medicine, Menoufia University, Menoufia, Egypt; 5 Faculty of Medicine, Al-Azhar University, Cairo, Egypt; 6 Faculty of Medicine, Al-Azhar University, Damietta, Egypt; 7 Faculty of Medicine, Alexandria University, Alexandria, Egypt; 8 University Hospitals Cleveland Medical Center, Case Western Reserve University, Cleveland, OH USA; 9 MetroHealth Medical Center, Case Western Reserve University, Cleveland Heights, OH USA; 10 Department of Cardiovascular Medicine, Mayo Clinic, Arizona, USA; 11 Department of Cardiology, Faculty of Medicine, Suez Canal University, Ismailia, Egypt

**Keywords:** Ultrathin-strut drug-eluting stent, DES, Percutaneous coronary intervention, Meta-analysis

## Abstract

**Background:**

Newer generation ultrathin strut stents are associated with less incidence of target lesion failure (TLF) in patients undergoing percutaneous coronary intervention (PCI) in the short term. However, its long-term effect on different cardiovascular outcomes remains unknown.

**Objectives:**

We aim to identify the effects of newer-generation ultrathin-strut stents vs. standard thickness second-generation drug-eluting stents (DES) on long-term outcomes of revascularization in coronary artery disease.

**Methods:**

We searched PubMed, Web of Science, Cochrane Library databases, and Scopus for randomized controlled trials (RCTs) and registries that compare newer-generation ultrathin-strut (< 70 mm) with thicker strut (> 70 mm) DES to evaluate cardioprotective effects over a period of up to 5 years. Primary outcome was TLF, a composite of cardiac death, target vessel myocardial infarction (TVMI) or target lesion revascularization (TLR). Secondary outcomes included the components of TLF, stent thrombosis (ST), and all-cause death were pooled as the standardized mean difference between the two groups from baseline to endpoint.

**Results:**

We included 19 RCTs and two prospective registries (103,101 patients) in this analysis. The overall effect on the primary outcome was in favor of second-generation ultrathin struts stents in terms of TLF at ≥ 1 year, ≥ 2 years, and ≥ 3 years (*P* value = 0.01, 95% CI [0.75, 0.96]), *P* value = 0.003, 95% CI [0.77, 0.95]), *P* value = 0.007, 95% CI [0.76, 0.96]), respectively. However, there was no reported benefit in terms of TLF when we compared the two groups at ≥ 5 years (*P* value = 0.21), 95% CI [0.85, 1.04]). Some of the reported components of the primary and secondary outcomes, such as TLR, target vessel revascularization (TVR), and TVMI, showed the same pattern as the TLF outcome.

**Conclusion:**

Ultrathin-strut DES showed a beneficial effect over thicker strut stents for up to 3 years. However, at the 5-year follow-up, the ultrathin strut did not differ in terms of TLF, TLR, TVR, and TVMI compared with standard-thickness DES, with similar risks of patient-oriented composite endpoint (POCE), MI, ST, cardiac death, and all-cause mortality.

**Supplementary Information:**

The online version contains supplementary material available at 10.1186/s40001-024-01949-7.

## Introduction

Percutaneous coronary intervention (PCI) is the recommended revascularization approach for restoring blood flow to the heart in patients with stable coronary artery disease (SCAD) when medical treatment fails to enhance prognosis or alleviate symptoms (chest pain, weakness, short of breath) [1]. Additionally, it is the recommended reperfusion strategy for patients presenting with acute ST-segment elevation myocardial infarction (STEMI) [2]. The implementation of first-generation drug-eluting stents (DES) decreased the occurrence of restenosis compared to bare metal stents. However, this advancement was at the expense of higher rates of stent thrombosis (ST). The incidence of definite very late ST ranges from 0.6 to 0.7% per year, while the rate of major adverse cardiac events (MACE) showed a steady increase of 2.6% annually [3]. The occurrence of unfavorable outcomes with the first-generation and contemporary permanent polymer-based DES provides a chance for step-by-step enhancement [4–9].

Improved stent design, enhanced polymer coating, and the rate of release of antiproliferative agents have contributed to DES’s increased safety and efficacy. Second-generation thin-strut DES have demonstrated a reduced risk of restenosis, ST, myocardial infarction (MI), or even death compared to older-generation DES or bare metal stents [10, 11]. Additionally, newer generations of stents with ultrathin strut thickness or biodegradable polymers can accelerate endothelialization, enhance healing, reduce inflammation and arterial injury, and decrease neointimal proliferation and thrombogenicity [12].

Recent research showed that ultrathin-strut DES with a thickness of less than 70 µm can enhance outcomes even more than second-generation DES [13]. Ultrathin second-generation DES has been found to have lower rates of target lesion failure (TLF) at both 2 years and 3 years compared to second-generation DES with standard thickness, as demonstrated by a recent meta-analysis [14]. Nevertheless, the long-term safety and efficacy of the initial advantages granted by ultrathin second-generation DES is still unknown. Hence, we conducted an updated systematic review and meta-analysis, with an extended follow-up period of 5 years, to compare the clinical outcomes between ultrathin-strut and standard thickness second-generation DES.

## Methods

### Data collection and extraction

We searched PubMed, Scopus, Web of Science, and Cochrane Library databases up to November 2023 using the search terms: (Ultrathin strut OR Thin strut OR Orsiro stent) AND (Sirolimus-eluting stent OR SES OR drug-eluting stents OR DES) AND (Coronary artery intervention OR Percutaneous coronary intervention OR Coronary angioplasty OR Stent implantation).

Endnote software (Clarivate Analytics, PA, USA) removed duplicates. The retrieved references were screened in two steps: the first consisted of screening the titles/abstracts independently by (A.M, M.N, and A.H) to determine their relevance, and the second consisted of screening the full-text articles of the identified abstracts for final eligibility to the quantitative analysis. The Rayyan website was used in the selection process [15].

**Fig. 1:**
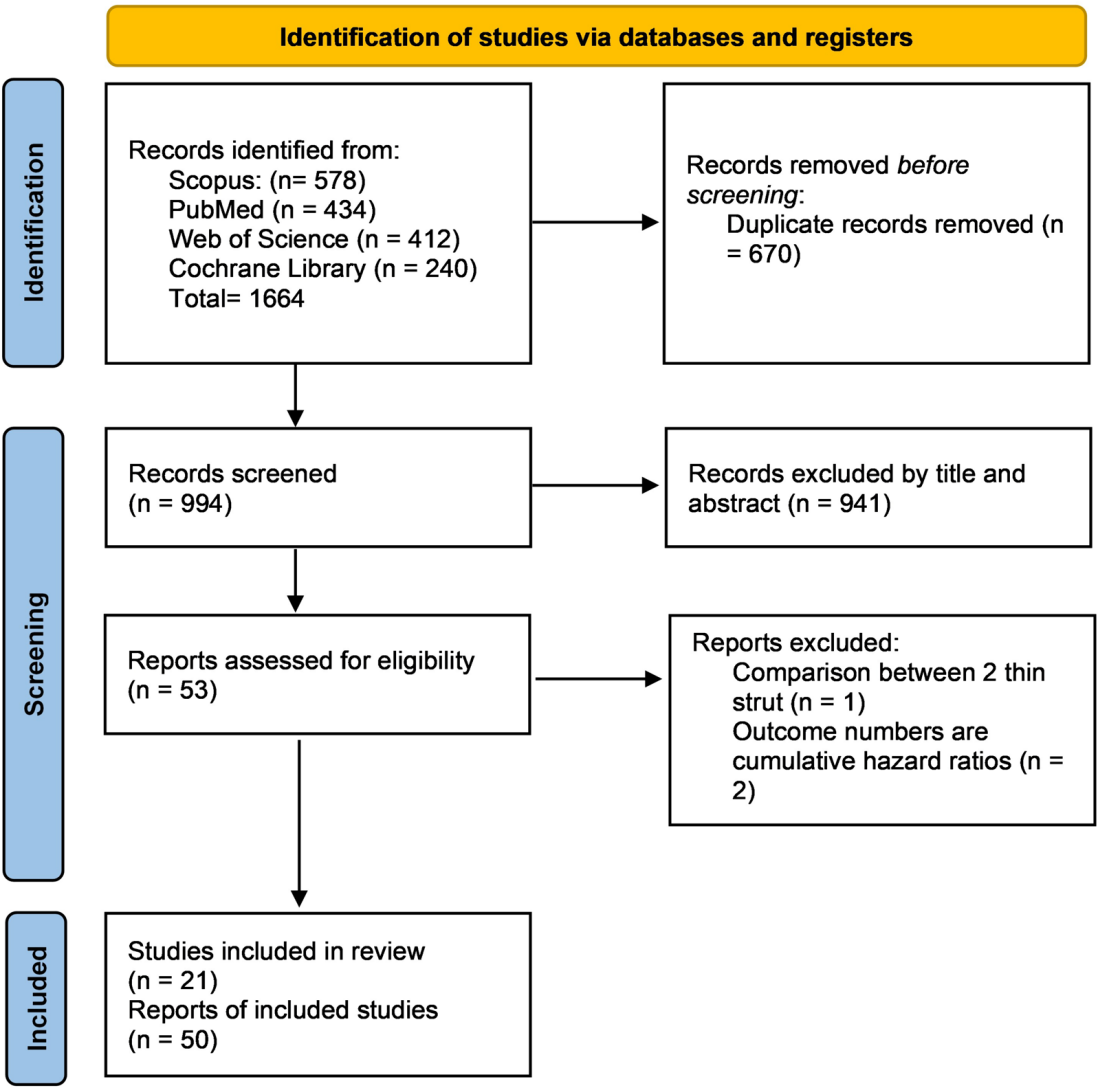
PRISMA flow diagram of the study

Our search identified 994 results after duplicates were removed. Following the title and abstract screening, 53 papers were selected for full-text review. Of them, 50 studies were included in the meta-analysis. No further papers were included after manually searching the references of the included studies. The selection process is illustrated in the PRISMA flow diagram of the study in Fig. 1 and was registered on PROSPERO (CRD42024506460).

Studies enrolled patients with coronary artery disease undergoing PCI, comparing ultrathin sirolimus-eluting stent vs. standard thickness second-generation DES in RCTs, and registries reporting clinical outcomes were included in our meta-analysis. Animal studies, non-English studies, abstracts without available data, and unpublished studies were excluded. The data were extracted to a uniform standardized data extraction sheet, including (1) a summary of study characteristics, (2) stent characteristics, (3) baseline patient characteristics, (4) lesion characteristics and treatment procedures, and (5) clinical outcomes.

### Outcomes

The primary endpoints of the current analysis included TLF, a composite of cardiac death, target vessel myocardial infarction (TVMI), and target lesion revascularization (TLR). Secondary outcomes included patient-oriented composite endpoint (POCE) of all-cause death, MI, repeat revascularization, and each component of TLF and ST. All outcomes are up to 5 years of follow-up.

### Risk-of-bias assessment

We utilized the revised Cochrane risk-of-bias tool for RCTs (RoB 2) to evaluate the risk of bias in the included clinical trials [16]. This evaluation encompassed an assessment of the randomization process, concealment of the allocation sequence, deviations from the intended interventions, utilization of appropriate analysis to estimate the effect of assignment to intervention, measurement of the outcome, selection of the reported results, and overall risk of bias. The assessment of the methodological quality of the studies was classified as either low risk, with some concerns, or high risk of bias. For prospective registries, we used The Cochrane ROBINS‐I tool [17], which includes the following domains: (1) bias due to confounding, (2) bias in the selection of participants into the study, (3) bias in the classification of interventions, (4) bias due to deviations from intended interventions, (5) bias due to missing data, bias in the measurement of outcomes, and (6) bias in the selection of the reported result. Any conflicts between the reviewers were resolved by consensus or consultation.

### Statistical analysis

We used RevMan v5.3 to conduct the statistical analysis [18]. We used the risk ratio (RR) to pool the results of dichotomous outcomes, and we used the mean difference (MD) with a 95% confidence interval (CI) to pool the continuous outcomes. We used the fixed-effects model. However, the random-effects model was used in case of significant heterogeneity. Chi-square and I-square tests were used to evaluate heterogeneity, where the Chi-square test detects the presence of heterogeneity, and the I-square test evaluates its degree. I-square was interpreted in accordance with the Cochrane Handbook (chapter nine) [19] as follows: heterogeneity is not significant for 0–40%, moderate for 30–60%, substantial for 50–90%, and considerable for 75–100%. We considered an alpha level below 0.1 for the Chi-square test to detect significant heterogeneity. We performed a leave-one-out sensitivity analysis to address the heterogeneity in our pooled studies. By systematically excluding each study one at a time, we identified which studies contributed to the heterogeneity and reported our findings accordingly. We used Stata MP version 17 (Stata Corp) to assess the publication bias by inspection and Egger’s test in outcomes reported by ten or more studies. We conducted a subgroup analysis for the follow-up duration as follows: ≥ 1 year (any study’s follow-up duration from 1 year to less than 2 years), ≥ 2 years (any study’s follow-up duration from 2 years to less than 3 years), ≥ 3 years (any study’s follow-up duration from 3 years to less than 4 years), and ≥ 5 years (any study’s follow-up duration 5 years or more).

We conducted a subgroup analysis comparing acute coronary syndrome (ACS) and chronic coronary syndrome (CCS) patients for all available outcomes across all follow-up durations. We detected a subgroup difference using the test of subgroup difference.

## Results

**Fig. 2:**
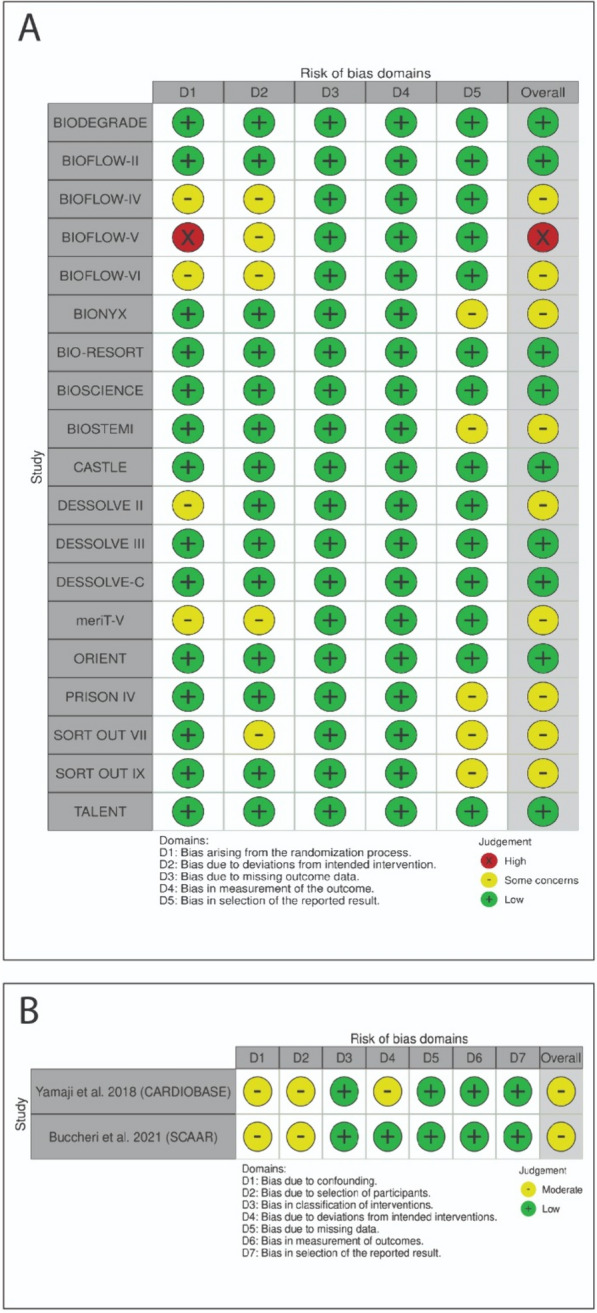
Risk of bias and quality assessment

After a detailed search, 19 RCTs and two registries were included in our meta-analysis [20–69], according to the Cochrane RoB2 and ROBINS-1 assessments. Nine studies had an overall low risk of bias, 11 had some concerns, and one had an overall high risk of bias (Fig. 2). Analysis of publication bias is summarized in Supplementary Table 3.

### Characteristics of the included studies

These studies included 103,101 patients who underwent PCI for coronary artery disease (for both CCS and ACS) using ultrathin-struts DES, *n* = 19,001; standard thickness second-generation DES, *n* = 84,100). Nine studies have reached five 5-year follow-ups, five studies have reached three 5-year follow-ups, three studies have reached 2-year follow-ups, and 4 years have reached 1-year follow-ups. The details of studies characteristics are presented in Table S2. Summary of stent characteristics, baseline patient characteristics, lesion characteristics, and intervention procedures of the are outlined in Tables 1, 2, and 3.

**Table 1:** Summary of stent characteristics

Stent	Strut thickness	Stent platform	Drug eluted	Timing of drug elution	Coating polymer	Timing of polymer degradation
Orsiro	60 µm	Cobalt–chromium	Sirolimus	12–14 weeks	Biodegradable polymer made of poly-l-lactic acid	12–24 months
Xience Sierra and Xpedition	81 µm	Cobalt–chromium	Everolimus		Durable polymer/nonerasable polymer made of polyvinylidene fluoride–hexafluoropropylene	24 months
TIVOLI	80 µm	Cobalt–chromium (L605)	Sirolimus	75% at 28 days	Biodegradable polymer PLGA	–
BioMatrix	120 μm	Stainless steel platform	Biolimus	6 months	Polylactic acid albumin-l polymer that is degradable	9 months
BioFreedom	120 μm	Stainless steel, a polymer-free and carrier-free drug-coated stent	Biolimus	90% of drug within 48 h	Polymer-free and carrier-free drug-coated stent. The stent transfers umirolimus (also known as biolimus A9), a highly lipophilic sirolimus analogue (15.6 μg/mm^2^) into the vessel wall over a period of 1 month	–
Supraflex	60 μm	L605 cobalt–chromium	Sirolimus	48 days	Biodegradable polymeric matrix coating (poly l-lactide, 50:50 mixture poly d,l-lactide-*co*-glycolide and polyvinyl pyrrolidone)	9–12 months
Resolute Onyx	81/91 µm 2	Cobalt–chromium, platinum–iridium core wire	Zotarolimus	6 months	Covered with a 5·6 μm layer of the BioLinx durable polymer	–
MiStent	64 μm	Cobalt–chromium	Microcrystalline sirolimus	9 months	Biodegradable polylactic-coglycolic	90 days
Nobori	120 μm	Stainless steel and a nondegradable parylene coating between the stent and the biodegradable polymer	Biolimus	≤ 30 days	The biodegradable polylactic acid polymer (PLLA and poly d,l-lactide-*co*-glycolide)	6–9 months
Endeavor	91 μm	Chromium–cobalt–nickel alloy	Zotarolimus	–	Durable phosphorylcholine polymer	–

**Table 2:** Baseline characteristics

Name of trial or registry	Age, y, mean (SD)		Men, *n* (%)		BMI, kg/m^2^, mean (SD),		DM, *n* (%)		HTN, *n* (%)		Dyslipidemia, *n* (%)		Current smoker, *n* (%)		Previous MI, *n* (%)		Previous PCI, *n* (%)		Previous CABG, *n* (%)	
	I	C	I	C	I	C	I	C	I	C	I	C	I	C	I	C	I	C	I	C
DESSOLVE-C [20]	59.55 ± 9.21	60.39 ± 8.62	148 (68.52)	137 (64.62)	24.78 ± 3.37	25.09 ± 3.31	53 (24.54)	57 (26.89)	122 (56.48)	119 (56.13)	29 (13.43)	26 (12.26)	94 (43.52)	91 (42.92)	23 (10.65)	35 (16.51)	–	–	0 (0%)	1 (0.47)
CASTLE [21]	70.1 ± 10.4	70.4 ± 10.1	572 (79.2)	554 (77.2)	–	–	284 (39.3)	279 (38.9)	478/722 (66.2)	491 (68.4)	467 (64.7)	439 (61.1)	128 (17.7)	138 (19.2)	122 (16.9)	107 (14.9)	275 (38.1)	255/718 (35.5)	24 (3.3)	18 (2.5)
SCAAR [22]	67.2 ± 11.1	67.8 ± 10.9	3378 (74.1)	51,296 (73.7)	–	–	984 (22.1)	14,782 (21.4)	2799 (62.9)	42,283 (61.5)	2087 (47)	33,355 (48.6)	911 (21.9)	13,067 (19.7)	963 (22.1)	14,337 (21)	778 (17.1)	12,370 (17.8)	367 (8.1)	5879 (8.5)
BIODEGRADE [23, 24]	63.4 ± 10.7	63.6 ± 11.1	835 (71.6)	838 (72.2)	25.1 ± 3.3	25.1 ± 3.3	384 (32.9)	393 (33.9)	685 (58.7)	706 (60.9)	609 (52.2)	625 (53.9)	324 (27.8)	306 (26.4)	60 (5.1)	56 (4.8)	135 (11.6)	147 (12.7)	8 (0.7)	10 (0.9)
SORT OUT IX [25, 26]	66.1 ± 11.1	66.4 ± 10.7	1,221 (77.3)	1,219 (77.5)	27.6 ± 8.0	27.8 ± 7.5	303 (19.2)	304 (19.3)	850 (56.0)	850 (56.0)	777 (51.5)	830 (55.0)	437 (29.3)	443 (29.8)	234 (15.2)	224 (14.7)	311 (20.9)	322 (20.9)	108 (7.0)	130 (8.4)
BIOFLOW VI [27]	59.1 ± 8.5	58.4 ± 8.6	160 (72.7)	142 (64.5)	25.3 ± 3.1	25.1 ± 2.8	60 (27.3)	58 (26.4)	120 (54.5)	125 (56.8)	84 (38.2)	93 (42.3)	75 (34.1)	81 (36.8)	31 (14.1)	17 (7.7)	0 (0)	0 (0)	–	–
BIOSTEMI [28–30]	62·2 (11·8)	63·2 (11·8)	513 (79)	477 (73)	26·9 (4·3)	26·8 (4·3)	73 (11%	82 (13%)	281 (43)	297 (46)	304 (47)	302/644 (47)	294 (45)	250/635 (39)	27 (4%)	24 (4%)	29 (4%)	34 (5%)	2 (< 1%)	8 (1)
BIOFLOW-IV [31, 32]	64.8 ± 9.6	64.4 ± 9.8	280 (72.2)	146 (76.8)	–	–	117 (30.4)	59 (31.1)	296 (76.9)	136 (71.6)	261 (67.8)	136 (71.6)	82 (21.3)	53 (27.9)	114 (29.6)	62 (32.6)	169 (43.9)	88 (46.3)	–	–
TALENT [33–35]	65.3 ± 10.4	65.3 ± 10.4	546 (75·8)	547 (76·5)	28·3 ± 4·8	28·3 ± 4·6	157 (21·8)	178 (24·9)	470 (65·3)	472 (66·1)	444 (61·8)	428 (60·2)	176 (24·5)	172 (24·1%)	136 (18·9)	128 (17·9)	175 (24·3)	153 (21·4%)	33 (4·6%)	55 (7·7)
MeriT-V [36, 37]	64.33 ± 9.57	64.70 ± 8.99	111 (65.29)	53 (61.63)	28.64 ± 4.45	29.40 ± 4.39	41 (24.12	18 (20.93)	125 (73.53)	68 (79.07)	118 (69.41)	59 (68.60)	71 (41.76)	41 (47.67)	37 (21.76)	13 (15.12)	31 (18.24)	14 (16.28)	–	–
BIONYX 3-year [38–40]	63·9 ± 11·2	64·1 ± 10·9	948 (76.1)	946 (76.1)	28·0 ± 4·4	27·9 ± 4·4	250 (20·1)	260 (20·9)	651/1223 (53·2)	611/1228 (49·8)	562/1212 (46·4)	552/1215 (45·4)	370/1204 (30·7)	371/1214 (30·6)	206 (16·5)	194 (15·6)	278 (22·3)	262 (21·1)	97 (7·8)	79 (6·4)
DESSOLVE III [41–43]	66.4 ± 10.7	66.3 ± 10.7	494 (70.3)	513 (73.8)	27.9 ± 4.4	28.1 ± 4.5	186 (26.6)	187 (27.2)	496 (71.5)	517 (75.4)	408 (61%)	393 (60%)	171 (26.6)	168 (26.4)	190 (27.1)	192 (27.8)	236 (33.7)	247 (35.6)	51 (7.3)	66 (9.5)
CARDIOBASE Bern PCI Registry [44]	67.7 ± 11.8	67.6 ± 12.1	1076 (74.2)	1064 (73.3)	27.5 ± 4.6	27.6 ± 4.8	328 (22.6)	341 (23.5)	1029 (70.9)	1030 (71.0)	981 (67.6)	971 (66.9)	397 (27.4)	399 (27.5)	218 (15.0)	219 (15.1)	317 (21.8)	327 (22.5)	149 (10.3)	148 (10.2)
ORIENT [45, 46]	65.2 ± 11.9	64.8 ± 11.0	180 (72.0)	86 (70.5)	24.8 ± 3.5	24.5 ± 3.1	63 (25.2)	33 (27.0)	162 (64.8)	81 (66.4)	134 (53.6)	66 (54.1)	66 (26.4)	35 (28.7)	–	–	34 (13.6)	18 (14.8)	2 (0.8)	0 (0.0)
PRISON IV [47–49]	62.4 ± 10.5	62.8 ± 9.5	122 (73.9)	137 (83.0)	–	–	31 (18.8)	34 (20.6)	148 (89.7)	154 (93.3)	161 (97.6)	155 (93.9)	49 (29.7)	59 (35.8)	52 (31.5)	48 (29.1)	47 (28.5)	50 (30.3)	6 (3.6)	11 (6.7)
BIO-RESORT [50–53]	64·2 ± 10·7	64·0 ± 10·7	854 (73)	1693 (72.19)	27·4 ± 4·2	27·6 ± 4·2	211 (18)	413 (17.6)	550 (47)	1074 (45.79)	463 (40)	872 (37.18)	341 (30)	690 (29.4)	209 (18)	440 (18.67)	214 (18%)	412 (17.57)	–	–
SORT OUT VII [54–57]	66.1 ± 10.7	64.8 ± 10.8	945 (74.9)	951 (75.2)	27.5 ± 4.7	27.4 ± 4.4	236 (18.7)	235 (18.6)	713 (58.1)	699 (56.4)	711 (57.6	706 (56.4)	355 (29.1)	399 (32.5)	215 (17.4)	222 (17.8)	237 (19.0)	256 (20.4)	100 (8.0)	96 (7.6)
BIOFLOW V [58–61]	64.5 ± 10.3	64.6 ± 10.7	660 (74.67)	328 (72.8)	–	–	300/883 (34)	166/449 (37)	696 (79.7)	354 (80.5)	695/881 (79)	370/449 (82)	209 (23.6)	102 (22.7)	238 (27.4)	115 (25.9)	323/877 (37)	147/445 (33)	62 (7.1)	23 (5)
DESSOLVE I and II [62–64]	65.0 ± 10.4	65.1 ± 10.5	85 (69.1)	45 (73.8)	–	–	23 (19%)	12 (19.7)	87 (70.5%)	42 (68.9)	90 (72.7)	50 (81.7)	26 (21.5)	15 (25.4)	28 (23.1)	10 (16.4)	39 (30.9)	14 (22.9)	5 (4.1%)	2 (3.3)
BIOFLOW-II [65, 66]	62.7 ± 10.4	64.8 ± 9.2	233 (78.2)	115 (74.7)	–	–	84 (28.2)	44 (28.6)	231 (77.5)	119 (77.3)	202 (67.8)	113 (20.1)	–	–	90 (30.2)	31 (20.13)	128 (43.0)	55 (35.7)	–	–
BIOSCIENCE [67–69]	66·1 (11·6)	65·9 (11·4)	818 (77·0)	816 (77·3)	27·8 (4·5)	27·5 (4·5)	257 (24·2)	229 (21·7)	728 (68·5)	706 (66·9)	712 (67·0)	716 (67·8)	309 (29·1)	300 (28·5)	223 (21·0)	204 (19·3)	325 (30·6)	292 (27·7)	113 (10·6)	98 (9·3)

Name of trial or registry	Clinical diagnosis for percutaneous coronary I, *n* (%)										Target lesion per patient, *n* (%)										Follow-up
	Silent ischemia		Stable angina		Unstable angina		Non-STEMI		STEMI		1		2		3		> 3		Number per patient		
	I	C	I	C	I	C	I	C	I	C	I	C	I	C	I	C	I	C	I	C	
DESSOLVE-C [20]	1 (0.46)	1 (0.47)	27 (12.50)	22 (10.38)	141 (65.28)	146 (68.87)	–	–	–	–	–	–	–	–	–	–	–	–	1.39 ± 0.50	1.30 ± 0.46	1 year
CASTLE [21]	181 (24.9)	177 (24.7)	389 (53.9)	399 (55.6)	30 (4.2)	38 (5.3)	30 (4.2)	25 (3.5)	45 (6.2)	38 (5.3)	–	–	–	–	–	–	–	–	–	–	1 year
SCAAR [22]	–	–	932 (20.4)	14,493 (20.8)	442 (9.7)	6864 (9.9)	1765 (38.7)	27,590 (39.7)	1300 (28.5)	18,514 (26.6)	3012 (6)	39,147 (56.3)	1096 (24.0)	18,610 (26.8)	345 (7.6)	7362 (10.6)	108 (2.4)	4451 (6.4)	261 (5.7)	65,021 (93.5)	1 and 2 years
BIODEGRADE [23, 24]	55 (4.7)	65 (5.6)	328 (28.1)	313 (27.0)	424 (36.4)	424 (36.6)	238 (20.4)	257 (22.2)	121 (10.4)	101 (8.7)	854 (73.2)	848 (73.1)	246 (21.1)	236 (20.3)	62 (5.3)	61 (5.3)	8 (0.7)	14 (1.2)	1.3	1.3	18 months and 3 years
SORT OUT IX [25, 26]	–	–	645 (40.8)	671 (42.7)	453 (28.7)	–	–	454 (28.9)	397 (25.1)	367 (23.3)	1196 (75.7)	1209 (76.9)	311 (19.7)	282 (17.9)	59 (3.7)	67 (4.3)	13 (0.8)	14 (0.9)	1.3 (0.6)	1.3 (0.6)	1 and 2 years
BIOFLOW VI [27]	–	–	–	–	161 (73.2)	184 (83.6)	–	–	–	–	186 (84.5)	189 (85.9)	34 (15.5)	31 (14.1)	–	–	–	–	1.15 (0.36)	1.14 (31)	1 Year
BIOSTEMI [28–30]	–	–	–	–	–	–	–	–	–	–	516 (80)	523 (80)	103 (16)	103 (16)	23 (4%)	23 (4%)	6 (1%)	2 (< 1%)	649	651	1, 2, and 5 Years
BIOFLOW-IV [31, 32]	–	–	–	–	–	–	–	–	–	–	–	–	–	–	–	–	–	–	–	–	1 and 5 years
TALENT [33–35]	–	–	291 (40·4)	310 (43·4)	116 (16·1)	99 (13·8)	194 (26·9)	189 (26·4)	119 (16·5)	117 (16·4)	–	–	–	–	–	–	–	–	–	–	1 and 5 years
MeriT–V [36, 37]	16 (9.41)	5 (5.81)	116 (68.24)	61 (70.9)	25 (14.71)	12 (13.95)	10 (5.88)	8 (9.30)	3 (1.76)	0 (0.0)	144 (84.71)	73 (84.88)	25 (14.71)	12 (13.95)	1 (0.59)	1 (1.16)	–	–	170	86	1, 2, and 3 years
BIONYX 3–year [38–40]	360 (28·9)	–	–	363 (29·2)	236 (19·0)	254 (20·4)	310 (24·9)	344 (27·7)	339 (27·2)	282 (22·7)	–	–	–	–	–	–	–	–	–	–	1 and 2 years
DESSOLVE III [41–43]	–	–	289 (41.1)	287 (41.3)	162 (23)	166 (23.9)	149 (21.2)	133 (19.1)	103 (14.7)	109 (15.7)	–	–	–	–	–	–	–	–	–	–	1,2, and 3 years
CARDIOBASE Bern PCI Registry [44]	–	–	672 (46.3%)	672 (46.3%)	76 (5.2%)	76 (5.2%)	382 (26.3)	382 (26.3)	321 (22.1)	321 (22.1)	799 (55.1)	824 (56.8)	429 (29.6)	411 (28.3)	223 (15.4)			216 (14.9)	–	–	1, 2, and 3 years
ORIENT [45, 46]	–	–	136 (53.3)	70 (55.1)	62 (24.3)	25 (19.7)	33 (12.9)	21 (16.5)	24 (9.4)	11 (8.7)	–	–	–	–	–	–	–	–	–	–	1 year
PRISON IV [47–49]	–	–	115 (69.7)	115 (69.7	10 (6.1)	12 (7.3)	–	–	–	–	105 (64.5)	108 (65.5)	50 (30.3)	46 (27.9)	10 (6.1)	11 (6.7)	–	–	165	165	1,2, and 3 years
BIO–RESORT [50–53]	–	–	351 (30%)	714 (30.45)	209 (18%)	411 (17.5)	239 (20%)	517 (22.05)	370 (32%)	703 (29.98)	–	–	–	–	–	–	–	–	–	–	1, 3, and 5 years
SORT OUT VII [54–57]	–	–	559 (44.3)	555 (43.9)	388 (30.7)			412 (32.6)	268 (21.2)	262 (20.7)	978 (77.6)	995 (78.7)	240 (19.0)	215 (17.0)	41 (3.3)	46 (3.6)	2 (0.2)	7 (0.6)	1.3 (0.5)	1.3 (0.5)	1,2,3,4, and 5 years
BIOFLOW V [58–61]	109/884 (12)	61/449 (14)	428 (48.4)	213 (47.7)	347 (39.3)	175 (39)	–	–	–	–									1.2 (0.4)	1.3 (0.5)	1,2,3, and 5 years
DESSOLVE I and II [62–64]	–	–	96 (78%)	49 (80%)	18 (14.6)	8 (13.3%)	–	–	–	–	–	–	–	–	–	–	–	–	–	–	9 months, 2, and 5 years
BIOFLOW–II [65, 66]	68 (22.6)	39 (24.7)	173 (57.9)	92 (59.7)	59 (19.5)	25 (15.6)	–	–	–	–	–	–	–	–	–	–	–	–	–	–	1 and 5 years
BIOSCIENCE [67–69]	161 (15·1%)	171 (16·2%)	325 (30·6%)	331 (31·3%)	78 (7·3%	74 (7%)	288 (27·1%)	284 (26·9)	211 (19·9)	196 (18·6)	683 (64·3)	688 (65·2)	266 (25·0)	267 (25·3)	84 (7·9)	86 (8·1%)	30 (2·8%)	15 (1·4%)	1594	1545	1, 2, and 5 Years

Data presented as mean and SD, or number (%)

Intervention (I), control (C)

*BMI* Body mass index, *CABG* coronary artery bypass graft, *DM* diabetes mellitus, *HTN* hypertension, *MI* myocardial infarction, *PCI* percutaneous coronary intervention, *STEMI* ST-segment elevation myocardial infarction

**Table 3:** Lesion characteristics and intervention procedure

Name of trial or registry	Target vessel location, *n* (%)								Lesion type, *n* (%)													
	Left main artery		Left anterior descending		Left circumflex artery		Right coronary artery		A		B1		B2		C		Chronic total occlusion		Bifurcation lesion		Direct stenting	
	I	C	I	C	I	C	I	C	I	C	I	C	I	C	I	C	I	C	I	C	I	C
DESSOLVE-C [20]	0 (0%)	0 (0%)	137 (47.24)	126 (45.99)	50 (17.24)	46 (16.79)	–	–	–	–	–	–	–	–	–	–	–	–	–	–	–	–
CASTLE [21]	–	–	449/845 (53.1)	458/841 (54.5)	129/845 (15.3)	147/841 (17.5)	269/845 (31.8)	236/841 (28.1)	–	–	–	–	625/811 (77.1)	–	–	615/811 (75.8)	–	–	267/845 (31.6)	269/841 (32.0)	–	–
SCAAR [22]	117 (2.6)	3821 (5.5)	2333 (51.2)	37,307 (53.6)	1264 (27.7)	20,206 (29.0)	1480 (32.5)	22,799 (32.8)	–	–	–	–	2993 (65.6)	–	–	42,860 (61.6)	229 (5)	3729 (5.4)	868 (19)	13,031 (18.7)	1019 (22.4)	17,860 (25.7)
BIODEGRADE [23, 24]	43 (2.8)	57 (3.8)	733 (48.2)	775 (51.3)	340 (22.4)	345 (22.9)	441 (29.0)	386 (25.6)	118 (7.8)	106 (7.0)	413 (27.2)	395 (26.2)	389 (25.6)	385 (25.5)	600 (39.5)	624 (41.3)	93 (6.1)	72 (4.8)	321 (15.2)	219 (14.5)	172 (11.3)	174 (11.5)
SORT OUT IX [25, 26]	44 (2.2)	49 (2.5)	856 (43.0)	845 (43.0)	445 (22.4)	465 (23.7)	621 (31.3)	589 (30.0)	216 (10.9)	211 (10.8)	612 (31.0)	561 (28.6)	503 (25.5)	526 (26.8)	645 (32.6)	662 (33.8)	81 (4.1)	100 (5.1)	407 (20.6)	368 (18.8)	194 (9.8)	160 (8.2)
BIOFLOW VI [27]	0 (0)	0 (0)	127 (50)	135 (53.8)	51 (20.1)	46 (18.3)	74 (29.1)	69 (27.5)	–	–	–	–	196 (77.5)	–	–	198 (78.9)	–	–	45 (17.8)	47 (18.7)	–	–
BIOSTEMI [28–30]	10 (1%)	9 (1)	316 (39)	357 (44)	143 (18)	137 (17)	346 (42)	302 (37)	–	–	–	–	–	–	–	–	1 (< 1%)	3 (< 1%)	101 (12)	115 (14)	–	–
BIOFLOW-IV [31, 32]	1 (0.2)	1 (0.5)	174 (39.5)	87 (40.7)	106 (24)	59 (27.6)	160 (36.3)	67 (31.3)	74 (17.3)	38 (18.2)	208 (48.7)	92 (44)	69 (16.2)	44 (21.1)	76 (17.8)	35 (16.8)	–	–	20 (4.5)	12 (5.6)	–	–
TALENT [33–35]	15 (1·4%)	16 (1·6)	468 (44·7)	432 (41·9)	220 (21·0)	237 (23·0)	338 (32·3)	328 (31·8)	–	–	–	–	–	–	–	–	–	–	167 (16·0)	157 (15·2)	–	–
MeriT-V [36, 37]	–	–	86 (47.25)	32 (33.68)	37 (20.33)	27 (28.42)	59 (32.42)	36 (37.89)	15 (8.24)	12 (12.63)	50 (27.47)	25 (26.32)	60 (32.97)	24 (25.26	57 (31.32)	34 (35.79)	–	–	–	–	–	–
BIONYX 3–year [38–40]	–	–	54 (44.3%)	22 (36.1)	26 (21.3)	23 (37.7)	42 (34.4)	16 (26.2)	10 (8.2)	6 (9.8)	56 (45.9)	28 (45.9)	36 (29.5)	16 (26.2%)	20 (16.4)	11 (18%)	–	–	–	–	–	–
DESSOLVE III [41–43]	16 (1.5%)	14 (1.4)	430 (41.5%)	394 (39.7)	271 (26.1)	259 (26.1)	314 (30.3)	317 (31.9)	–	–	–	–	–	–	–	–	–	–	77 (7%)	69 (7%)	–	–
C ARDIOBASE Bern PCI Registry [44]	57 (2.4%)	59 (2.5)	1031 (42.9)	1010 (42.7)	581 (24.1)	574 (24.2)	679 (28.2)	664 (28.0)	–	–	–	–	–	–	–	–	–	–	341 (14.2%)	344 (14.5)	704 (30.6)	671 (29.7)
ORIENT [45, 46]	20 (5.8)	5 (2.8)	158 (45.8)	85 (48.3)	93 (27.0)	36 (20.5)	74 (21.4)	50 (28.4)	–	–	–	–	–	–	–	–	31 (9.0)	11 (6.3)	79 (22.9)	42 (23.9)	–	–
PRISON IV [47–49]	–	–	48 (29.1)	50 (30.3)	–	–	94 (57.0)	87 (52.7)	–	–	–	–	–	–	–	–	–	–	–	–	–	–
BIO–RESORT [50–53]	23 (2%)	53 (1.7)	679 (44%)	1204 (38.68)	338 (22%)	753 (24.19)	485 (31%)	1045 (33.58)	75 (5%)	150 (4.8)	332 (22%)	731 (23.5)	624 (40)	1202 (38.6)	514 (33%)	1017 (32.68)	52 (3%)	99 (3.18)	443 (29)	884 (28.4)	–	–
SORT OUT VII [54–57]	18 (1.1)	12 (0.8)	686 (43.1)	672 (42.3)	338 (21.3)	349 (22.0)	526 (33.1)	536 (33.8)	217 (13.6)	203 (12.8)	470 (29.6)	493 (31.0)	358 (22.5)	343 (21.6)	545 (34.3)	549 (34.6)	63 (4.0)	65 (4.2)	192 (12.3)	198 (12.7)	221 (14.5)	211 (13.7)
BIOFLOW V [58–61]	–	–	431/1051 (41)	231/561 (41)	279/1051 (27)	146/561 (26)	341/1051 (32)	184/561 (33)	–	–	–	–	–	–	–	–	–	–	156/1051 (15)	84/561 (15)	–	–
DESSOLVE I and II [62–64]	–	–	55 (44.3)	23 (61)	27 (21.3)	23 (37.7)	43 (34.4)	16 (26.2)	8.2	9.8	45.9	45.9	29.5	26.2	16.4	18	–	–	–	–	–	–
BIOFLOW–II [65, 66]	1 (0.30)	0 (0.00)	148 (44.71)	69 (39.88)	73 (22.05)	55 (31.79)	109 (32.93)	49 (28.32)	–	–	–	–	–	–	–	–	–	–	–	–	–	–
BIOSCIENCE [67–69]	29 (1·8)	28 (1.7)	649 (40·7)	679 (43·9)	370 (23·2)	341 (22·1)	505 (31·7)	452 (29·3)	–	–	–	–	–	–	–	–	278 (17·5)	253 (16·4)	262 (16·5)	260 (16·9)	428/1517 (28·2)	439/1483 (29·6)

Data presented as number (%)

Intervention (I), control (C)

### Primary outcome

#### Target lesion failure (TLF)

**Fig. 3:**
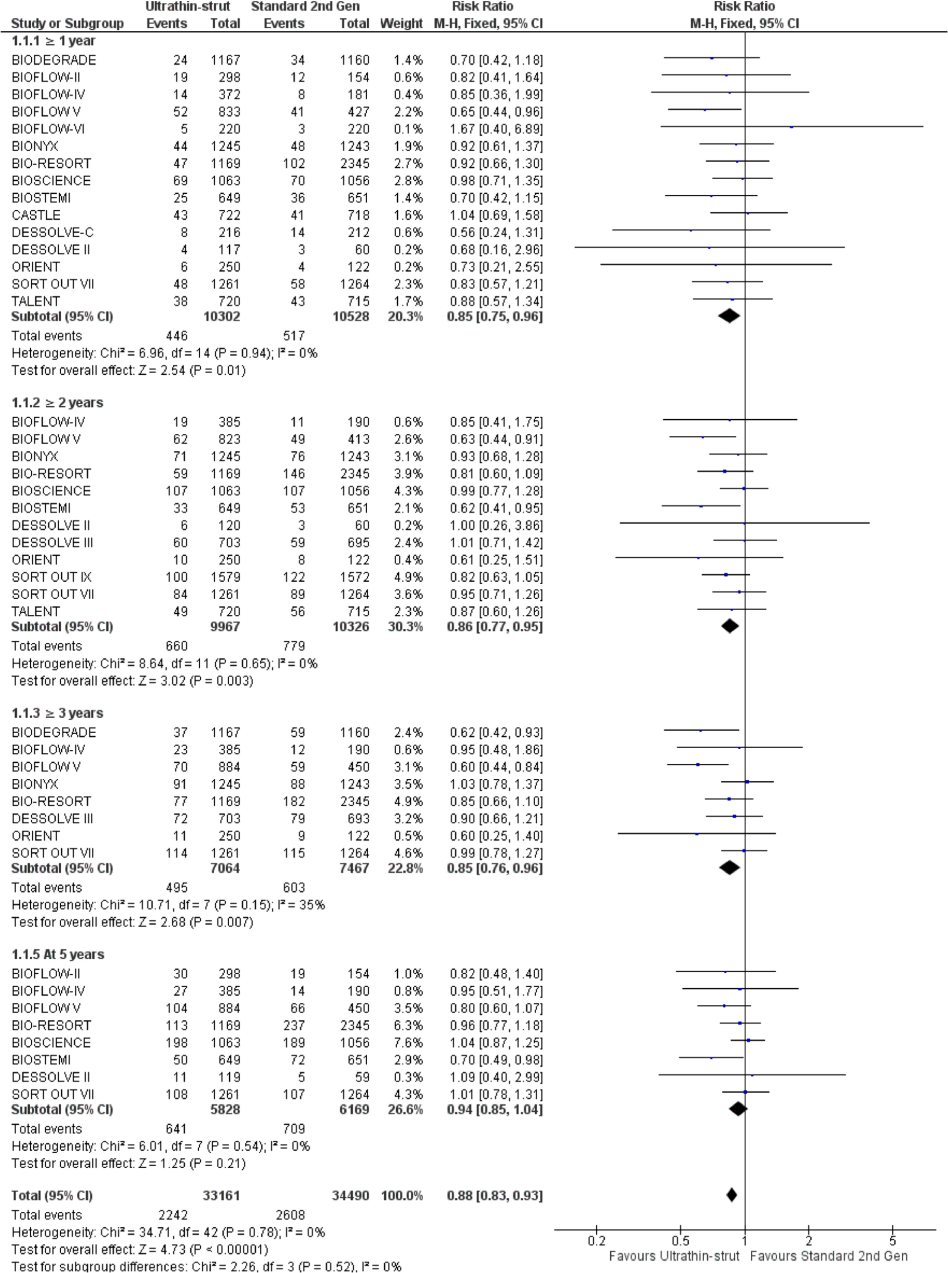
Forest plot of target lesion failure from 1 to 5 years follow-up

Ultrathin-struts DES were associated with a significant decreased in the incidence of TLF at ≥ 1 year (RR: 0.85 with 95% CI [0.75, 0.96], *P* = 0.01), at ≥ 2 years (RR: 0.86 with 95% CI [0.77, 0.95], *P* = 0.003), and at ≥ 3 years (RR: 0.85 with 95% CI [0.76, 0.96], *P* = 0.007) compared to standard thickness second-generation DES. However, there was no significant difference between ultrathin-struts DES and standard thickness second-generation DES at 5 years (RR: 0.94 with 95% CI [0.85, 1.04], *P* = 0.21) (Fig. 3).

#### Cardiac death

**Fig. 4:**
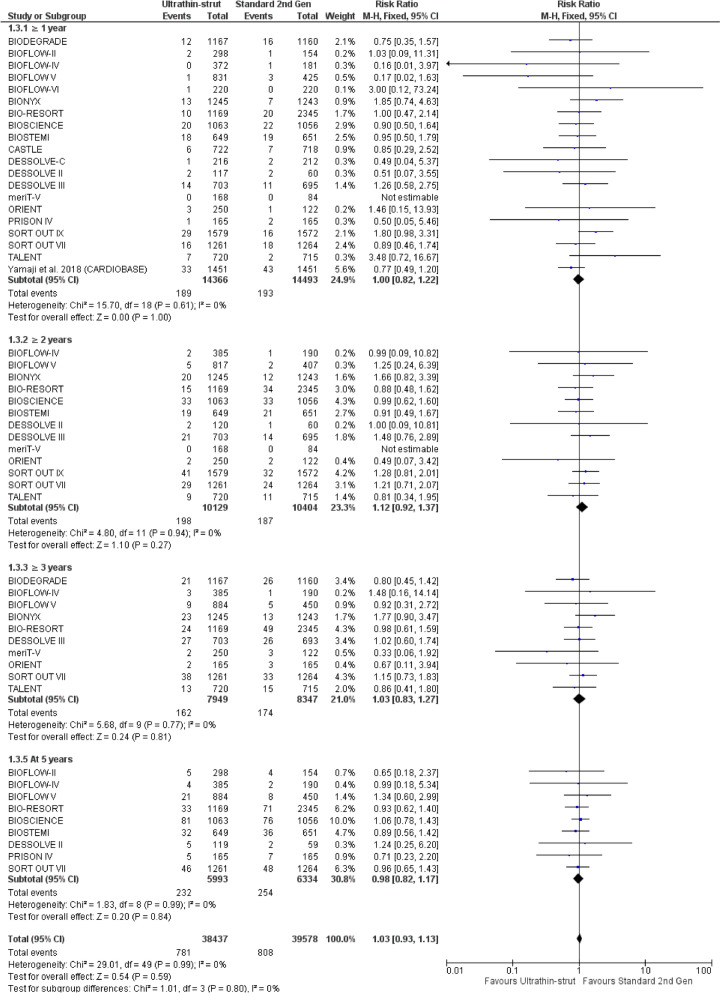
Forest plot of cardiac death from 1 to 5 years follow-up

There was no significant difference between ultrathin-struts DES and standard thickness second-generation DES at ≥ 1 year (RR: 1.00 with 95% CI [0.82, 1.22], *P* = 1.00), at ≥ 2 years (RR: 1.12 with 95% CI [0.92, 1.37], *P* = 0.27), at ≥ 3 years (RR: 1.03 with 95% CI [0.83, 1.27], *P* = 0.81), and at 5 years (RR: 0.98 with 95% CI [0.82, 1.17], *P* = 0.84) (Fig. 4).

#### Target vessel-related myocardial infarction (TVMI)

**Fig. 5:**
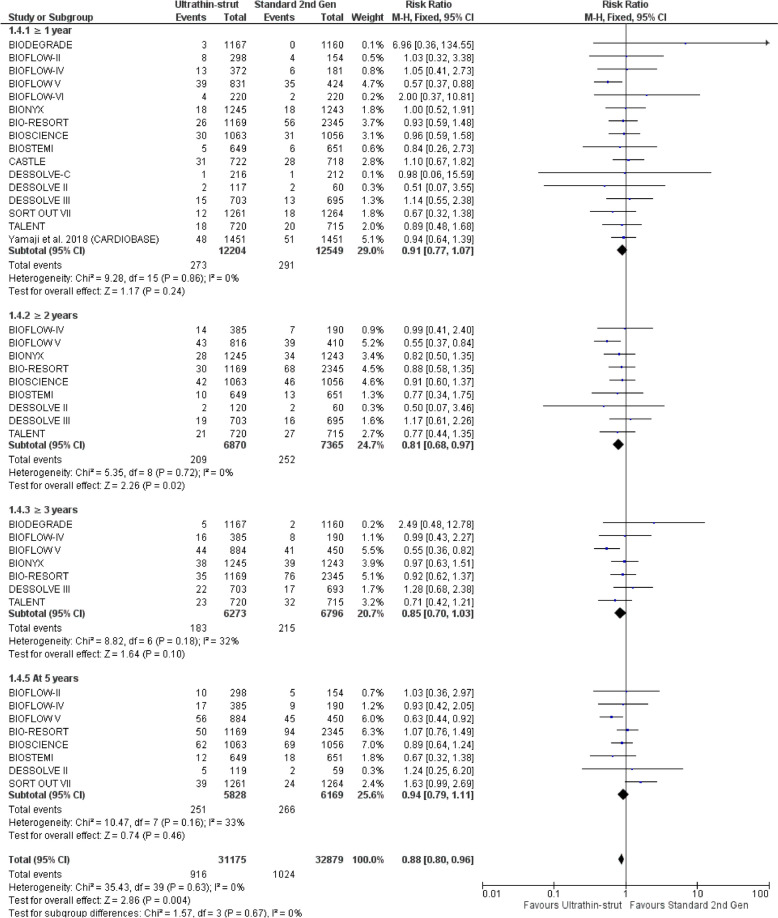
Forest plot of target vessel-related myocardial infarction (TVMI) from 1 to 5 years follow-up

Ultrathin-struts DES were associated with a decreased incidence of TVMI at ≥ 2 years (RR: 0.81 with 95% CI [0.68, 0.97], *P* = 0.02) compared to standard thickness second-generation DES, while there was no significant difference between ultrathin-struts DES and standard thickness second-generation DES at ≥ 1 year (RR: 0.91 with 95% CI [0.77, 1.07], *P* = 0.24), at ≥ 3 years (RR: 0.85 with 95% CI [0.70, 1.03], *P* = 0.10), and at 5 years (RR: 0.94 with 95% CI [0.79, 1.11], *P* = 0.46) (Fig. 5).

#### Target lesion revascularization (TLR)

**Fig. 6:**
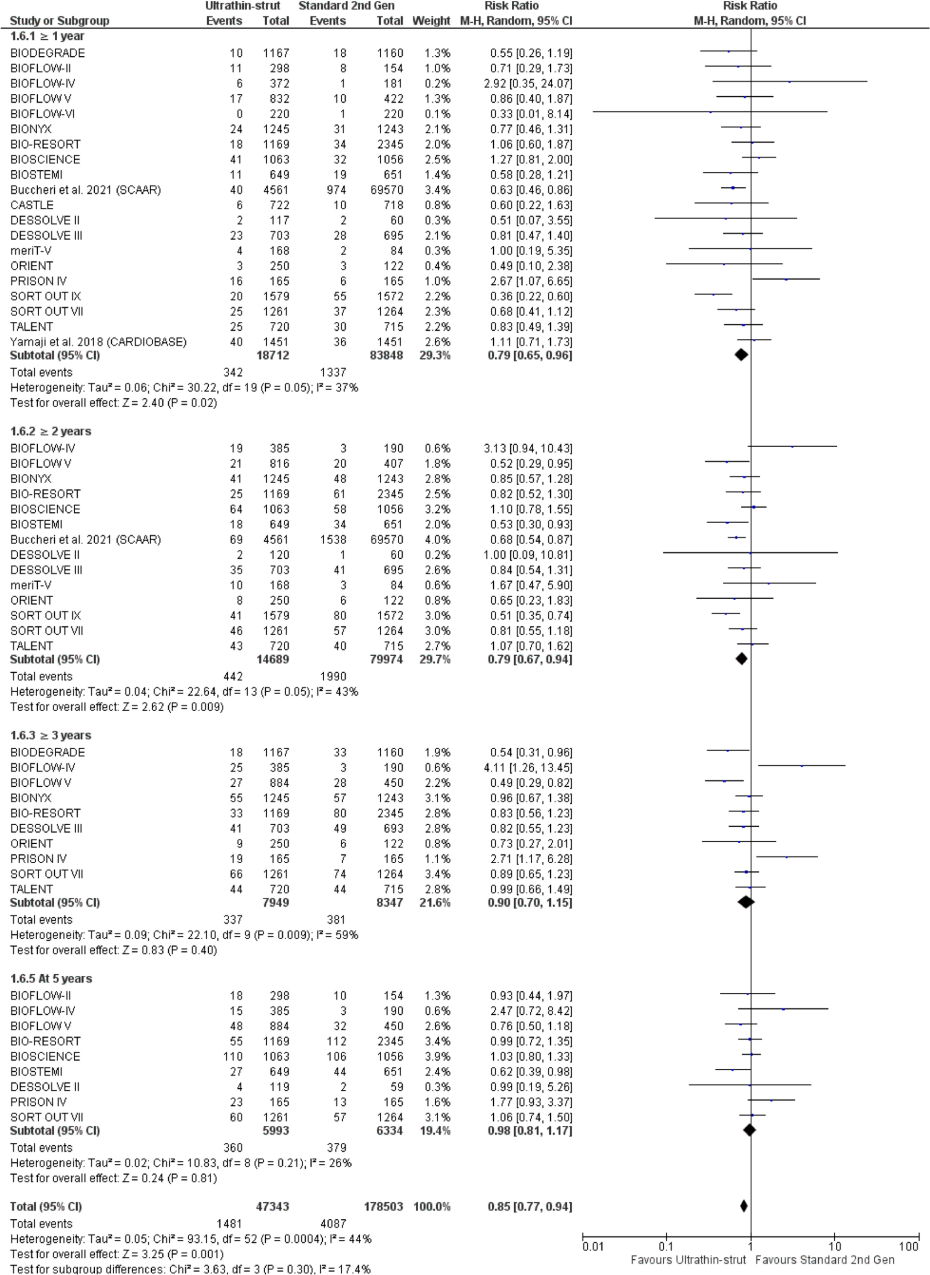
Forest plot of target lesion revascularization (TLR) from 1 to 5 years follow-up

Regarding the TLR, ultrathin-struts DES showed a lower incidence of TLR at ≥ 1 year (RR: 0.79 with 95% CI [0.65, 0.96], *P* = 0.02) and at ≥ 2 years (RR: 0.79 with 95% CI [0.67, 0.94], *P* = 0.009), compared to standard thickness second-generation DES. However, there was no significant difference between ultrathin-struts DES and standard thickness second-generation DES at ≥ 3 years (RR: 0.90 with 95% CI [0.70, 1.15], *P* = 0.40) and at 5 years (RR: 0.98 with 95% CI [0.81, 1.17], *P* = 0.81) (Fig. 6).

### Secondary outcome

#### Target vessel revascularization (TVR)

**Fig. 7:**
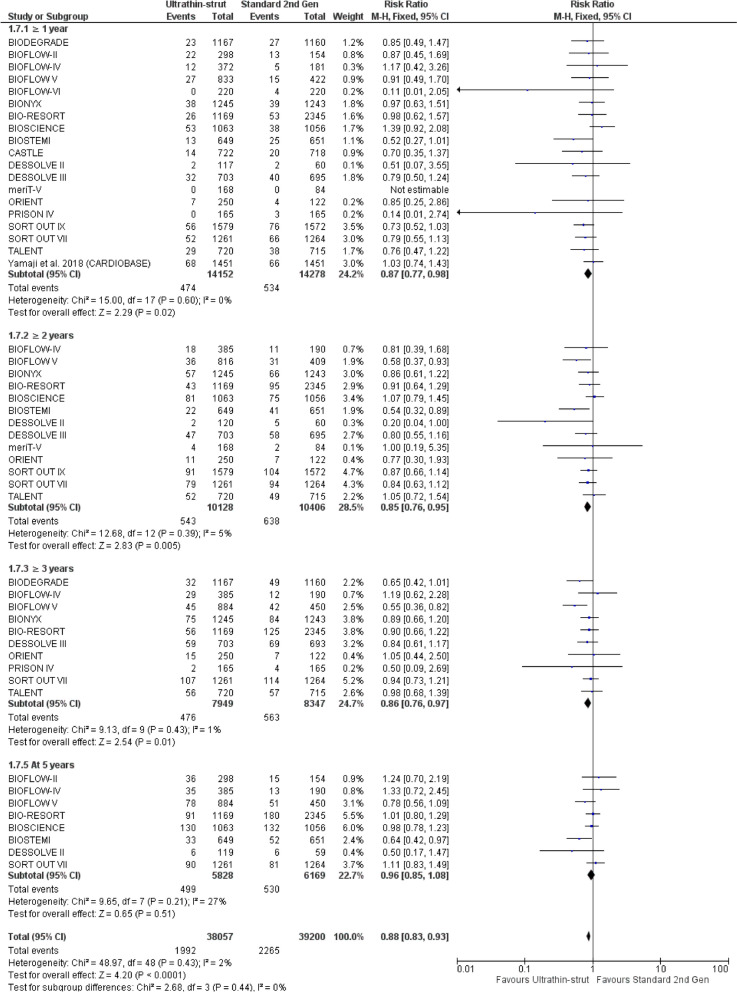
Forest plot of target vessel revascularization (TVR) from 1 to 5 years follow-up

The incidence of TVR was lower in ultrathin-struts DES TVR at ≥ 1 year (RR: 0.87 with 95% CI [0.77, 0.98], *P* = 0.02), at ≥ 2 years (RR: 0.85 with 95% CI [0.76, 0.95], *P* = 0.005), and at ≥ 3 years (RR: 0.86 with 95% CI [0.76, 0.97], *P* = 0.01) compared to standard thickness second-generation DES. There was no significant difference between ultrathin-struts DES and standard thickness second-generation DES at 5 years (RR: 0.96 with 95% CI [0.85, 1.08], *P* = 0.51) (Fig. 7).

**Fig. 8:**
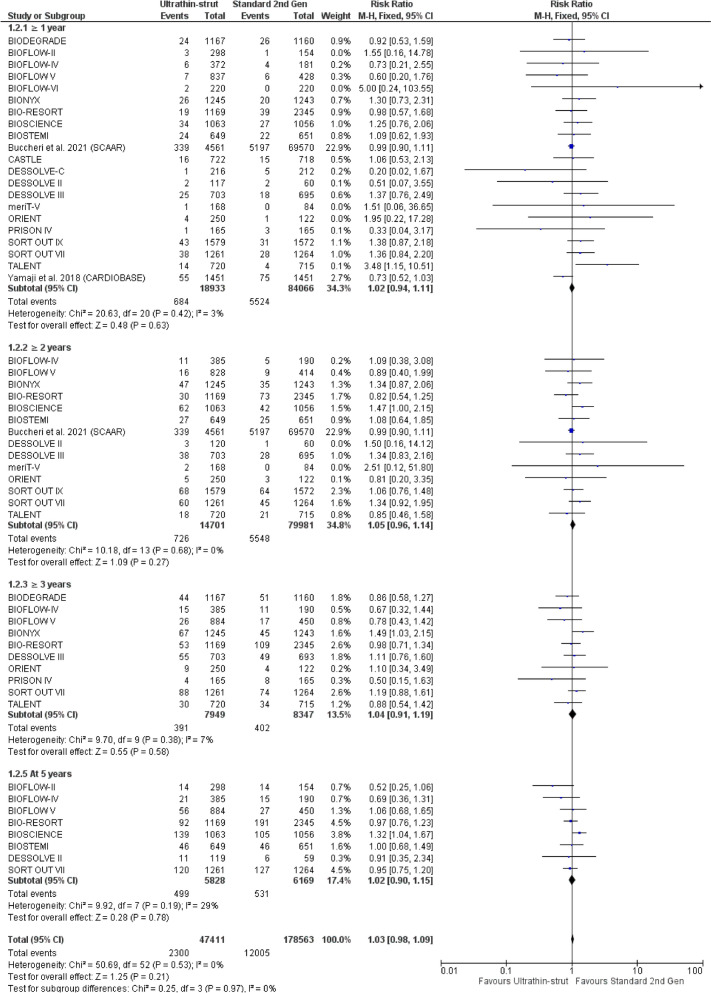
Forest plot of all-cause mortality from 1 to 5 years follow-up

There were no significant differences between ultra-thin-strut DES and standard thickness second-generation DES regarding all-cause mortality (Fig. 8), patient-oriented composite endpoint (POCE) (Figure S16), myocardial infarction (MI) (Figure S18), repeat revascularization (Figure S22), definite or probable stent thrombosis (ST) (Figure S24), definite stent thrombosis (ST) (Figure S27), probable stent thrombosis (ST) (Figure S29), and bleeding (Figure S30) at 1 year, ≥ 2 years, ≥ 3 years, and 5 years.

The details of primary and secondary outcome results are presented in Table 4.

**Table 4:** Summary of results analysis

Figure number	Outcomes	Subgroup	Total number of patients		Heterogeneity			Test for overall effect		Risk ratio	95%, CI
			Ultra-thin strut	Standard 2nd Gen	Chi2	d*f*	I2%	**Z (value)**	**P (value)**		
3	TLF	At ≥ 1 year	10,302	10,528	6.96	14 (*P* = 0.94)	0	2.54	0.01	0.85	[0.75,0.96]
		At ≥ 2 years	9976	10,326	8.64	11 (*P* = 0.65)	0	3.02	0.003	0.86	[0.77,0.95]
		At ≥ 3 years	7064	7467	10.71	7 (*P* = 0.15)	35	2.68	0.007	0.85	[0.76,0.96]
		At 5 years	5828	6169	6.01	7 (*P* = 0.54)	0	1.25	0.21	0.94	[0.85,1.04]
4	Cardiac death	At ≥ 1 year	14,366	14,493	15.7	18 (*P* = 0.61)	0	0	1	1	[0.82,1.22]
		At ≥ 2 years	10,129	10,404	4.8	11 (*P* = 0.94)	0	1.1	0.27	1.12	[0.92,1.37]
		At ≥ 3 years	7949	8347	5.68	9 (*P* = 0.77)	0	0.24	0.81	1.03	[0.83,1.27]
		At 5 years	5993	6334	1.83	8 (*P* = 0.99)	0	0.2	0.84	0.98	[0.82,1.17]
5	TVMI	At ≥ 1 year	12,204	12,549	9.28	15 (*P* = 0.86)	0	1.17	0.24	0.91	[0.77,1.07]
		At ≥ 2 years	6870	7365	5.35	8 (*P* = 0.72)	0	2.26	0.02	0.81	[0.68,0.97]
		At ≥ 3 years	6273	6796	8.82	6 (*P* = 0.18)	32	1.64	0.1	0.85	[0.70,1.03]
		At 5 years	5828	6169	10.47	7 (*P* = 0.16)	33	0.74	0.46	0.94	[0.79,1.11]
6	TLR	At ≥ 1 year	18,712	83,848	30.22	19 (*P* = 0.05)	37	2.4	0.02	0.79	[0.65,0.96]
		At ≥ 2 years	14,689	79,774	22.64	13 (*P* = 0.05)	43	2.62	0.009	0.79	[0.67,0.94]
		At ≥ 3 years	7949	8347	22.1	9 (*P* = 0.009)	59	0.83	0.4	0.9	[0.70,0.1.15]
		At 5 years	5993	6334	10.83	8 (*P* = 0.21)	26	0.24	0.81	0.98	[0.81,1.17]
7	TVR	At ≥ 1 year	14,152	14,278	15	17 (*P* = 0.60)	0	2.29	0.02	0.87	[0.77,0.98]
		At ≥ 2 years	10,128	10,406	12.68	12 (*P* = 0.39)	5	2.83	0.005	0.85	[0.76,0.95]
		At ≥ 3 years	7949	8347	9.13	9 (*P* = 0.43)	1	2.54	0.01	0.86	[0.76,0.97]
		At 5 years	5828	6169	9.65	7 (*P* = 0.21)	27	0.65	0.51	0.96	[0.83,0.94]
8	All-cause mortality	At ≥ 1 year	18,933	84,066	20.63	20 (*P* = 0.42)	3	0.48	0.63	1.02	[0.94,1.11]
		At ≥ 2 years	14,701	79,981	10.18	13 (*P* = 0.68)	0	1.09	0.27	1.05	[0.96,1.14]
		At ≥ 3 years	7949	9347	9.7	9 (*P* = 0.38)	7	0.55	0.58	1.04	[0.91,1.19]
		At 5 years	5828	6169	9.92	7 (*P* = 0.19)	29	0.28	0.78	1.02	[0.90,1.15]
S16	POCE	At ≥ 1 year	12,320	13,016	15.32	14 (*P* = 0.36)	9	0.42	0.67	1.02	[0.94,1.10]
		At ≥ 2 years	7756	8589	6.1	8 (*P* = 0.64)	0	0.22	0.82	1.01	[0.94,1.09]
		At ≥ 3 years	5655	6489	11.91	6 (*P* = 0.06)	50	1.01	1	1	[0.89,1.13]
		At 5 years	4825	5660	2.35	5 (*P* = 0.80)	0	0.81	0.42	1.03	[0.96,1.11]
S18	Any MI	At ≥ 1 year	18,928	84,063	23.98	20 (*P* = 0.24)	17	0.19	0.85	0.99	[0.89,1.10]
		At ≥ 2 years	15,596	79,979	11.41	13 (*P* = 0.58)	0	1.58	0.11	0.93	[0.85,1.02]
		At ≥ 3 years	7949	8347	11.84	9 (*P* = 0.22)	24	0.86	0.39	0.94	[0.80,1.09]
		At 5 years	5993	6334	8.66	8 (*P* = 0.37)	8	0.71	0.48	0.95	[0.83,1.09]
S22	Repeat revascularization	At ≥ 1 year	8648	9668	9.05	9 (*P* = 0.43)	1	0.19	0.85	0.99	[0.87,1.12]
		At ≥ 2 years	5795	6822	9.91	6 (*P* = 0.13)	39	0.46	0.65	0.97	[0.83,1.12]
		At ≥ 3 years	5250	6273	9.44	5 (*P* = 0.09)	47	0.94	0.35	0.93	[0.79,1.09]
		At 5 years	2881	4052	2.63	2 (*P* = 0.27)	24	0.83	0.41	0.94	[0.82,1.09]
S24	Definite or probable stent thrombosis	At ≥ 1 year	18,927	84,062	10.96	15 (*P* = 0.76)	0	0.86	0.39	0.92	[0.76,1.11]
		At ≥ 2 years	14,437	79,851	10.8	12 (*P* = 0.55)	0	1.54	0.12	0.86	[0.71,1.04]
		At ≥ 3 years	6367	6900	6.93	6 (*P* = 0.33)	13	0.16	0.87	0.98	[0.75,1.27]
		At 5 years	5828	6169	9.74	7 (*P* = 0.20)	28	1.53	0.13	0.83	[0.66,1.05]
S27	Definite stent thrombosis	At ≥ 1 year	10,969	11,570	11.46	9 (*P* = 0.25)	21	0.02	0.98	1	[0.72,1.40]
		At ≥ 2 years	9203	9947	8.35	4 (*P* = 0.40)	4	0.64	0.52	0.91	[0.67,1.23]
		At ≥ 3 years	5805	6606	7.97	5 (*P* = 0.16)	37	0.04	0.97	1.01	[0.66,1.54]
		At 5 years	4440	5470	4.46	4 (*P* = 0.35)	10	0.12	0.91	0.98	[0.70,1.38]
S29	Probable stent thrombosis	At ≥ 1 year	8815	9424	2.87	6 (*P* = 0.82)	0	1.31	0.19	0.76	[0.50,1.15]
		At ≥ 2 years	7958	8704	6.33	7 (*P* = 0.50)	0	1.61	0.11	0.76	[0.54,1.06]
		At ≥ 3 years	3391	4156	0.43	2 (*P* = 0.81)	0	0.46	0.65	1.24	[0.50,3.07]
		At 5 years	4440	5470	0.3	2 (*P* = 0.86)	0	0.44	0.66	0.92	[0.64,1.32]
S30	Bleeding	At ≥ 1 year	3847	3702	2.15	4 (*P* = 0.71)	0	0.39	0.7	1.06	[0.80,1.40]
		At ≥ 2 years	1958	1824	1.23	2 (*P* = 0.54)	0	0.16	0.88	0.97	[0.70,1.35]
		At ≥ 3 years	1413	1277	0.94	1 (*P* = 0.33)	0	0.09	0.93	1.02	[0.67,1.54]
		At 5 years	1712	1707	0.01	1 (*P* = 0.92)	0	0.63	0.53	1.1	[0.81,1.50]

TLF subgroup analysis regarding ACS versus CCS patients, there was no significant difference between ultrathin-struts DES and standard thickness second-generation DES at 1 year, 2 years, 3 years, and 5 years follow-up (*P* values for the subgroup analysis were 0.48, 0.97, 0.32, 0.63 consecutively) (Figures S31A–S31D).

More details about heterogeneity and sensitivity analysis are provided in the supplementary material.

## Discussion

In this systematic review and meta-analysis, which included 103,101 patients from 21 studies with 1- to 5-year follow-ups, we compared the safety and efficacy of ultrathin-struts DES to standard thickness second-generation DES, and we elucidated that

1. ultrathin struts have a lower incidence of TLF after 1, 2, and 3 years. Nevertheless, this benefit fades 5 years, with no noticeable difference.
2. At 1 and 2 years, ultrathin-struts DES showed a considerably decreased incidence of TLR compared to standard thickness second-generation DES. However, there is no significant difference in TLR between the two types of stents after 3 and 5 years.
3. No significant difference was noted between the two groups in terms of all secondary outcomes, except for TVR. The occurrence of TVR was lower in the ultrathin group during the initial 3-year period when compared with the group using thicker DES; nevertheless, this discrepancy disappeared at 5 years.

### Effect on outcomes components

One of the important components of the primary clinical outcomes is the TLF, which includes restenosis, thrombosis, and revascularization in the treated artery.

In our study, an ultrathin stent was associated with a lower incidence of TLF at 1, 2, 3 years, which could represent an early advantage and may be related to the short and intermediate-term effect of the ultrathin strut’s stents. On the other hand, at 5 years, the difference in TLF between the two types of stents was not noticeable, raising concerns about the long-term durability.

The positive effect of ultrathin stent in reducing the short and intermediate-term TLF may be attributable to the stent design. Ultrathin-struts DES have a unique design that differentiates them from the standard-thickness second-generation DES. The ultrathin strut design, measuring 60 µm, outperforms existing stents like XIENCE (81 µm) (Abbott Vascular, Santa Clara, CA) and RESOLUTE (91 µm) (Medtronic, Santa Rosa, CA, USA) in terms of flexibility and deliverability. This design reduces endothelial trauma, promoting excellent endothelial coverage and decreasing perivascular inflammation, resulting in a healthier vascular environment [70]. The ultrathin-strut DES evaluated in this meta-analysis has a similar metallic stent platform strut thickness and uses biodegradable polymers. They differ, however, in some elements of DES design, such as stent platform geometry, polymer composition, distribution or degradation time, and the kinetics of the antiproliferative medication delivered [12, 70]. Furthermore, characteristics inherent in the design, such as stent conformability and deliverability, can influence clinical outcomes in individuals with acute coronary syndromes (ACS), which offer a higher long-term sensitivity to stent-related adverse events. This is principally due to an enhanced prothrombotic and inflammatory response following the insertion of DES, leading to a delay in the healing process in the artery region where the stent is present [71]. Furthermore, the ultrathin design reduces side branch coverage even further, especially in vessels less than 3 mm in diameter, minimizing the risk of periprocedural myocardial infarction and, as a result, the incidence of TVMI [71].

The lack of a significant difference in all-cause mortality or even cardiac death between ultrathin DES and standard-thickness DES could be attributed to other contributing factors than the stent design, such as clinical, anatomical, and local pathophysiological lesion characteristics.

The findings of this study are consistent with previous research [13, 71, 72], showing that even minor changes in strut thickness, ranging from 20 to 30 mm, may be sufficient to produce unique stent-related outcomes in newer-generation DES in routine clinical settings. Our study’s effect on TLF aligns with the results of previously published meta-analyses [14, 72–74] except for Li et al., 2023 which showed no difference, and a smaller sample size can explain this. Our study is the first meta-analysis to compare the two groups regarding POCE and reported revascularization, and it showed no statistically significant difference between the two groups. Our results align with the previous meta-analyses [14, 72–74], which showed no statistically significant difference in all-cause mortality, cardiac mortality, and definite or probable ST outcomes.

The lower TLR in our study is contrary to the study by Madhavan [72], Monjur [73], Iglesias [74], and Li [75] results and in line with Hussain [14] which showed a significant reduction in TLR (RR, 0.85; 95% CI 0.72–1.00; *P* = 0.04) at 2 years.

Notably, while ultrathin-strut stents showed promising effects in the short term, their benefits may not be consistent over a more extended time. In our meta-analysis, there was no significant different in terms of TLF when we compared the two groups at ≥ 5 years. These findings might have a substantial implication on stent selection in clinical practice, particularly in patients at high risk of late and very late stent failure and requires more clinical trials to evaluate the long-term effect of ultrathin stent struts.

### Study limitations

This study has some limitations that affect the applicability of the study’s conclusions. The absence of specific patient data from the chosen trials limits the use of advanced statistical techniques, including multivariable and subgroup analyses, which hinders the investigation of variations in the initial characteristics between groups of patients receiving DES. Despite these drawbacks, the research offers insightful information about the state of research on ACS. The open-label design of the included studies presents possible confounders. This absence of blinding could introduce a potential source of bias by influencing intravascular imaging guiding and vessel preparation techniques between DES treatment groups. Also, the meta-analysis design has some intrinsic limitations, such as the reliance on aggregate study-level data, which limits the comparison depth compared to patient-level data. Patient-level analysis could enhance subgroup detection, providing a more nuanced understanding of the study outcomes. The SCAAR registry contributed to the large sample size of our study. This registry collected clinical data and procedural characteristics of all consecutive patients undergoing cardiac catheterization in Sweden, which may have influenced our overall results.

## Conclusion

This meta-analysis showed the non-inferiority of ultrathin stent DES compared to standardized thickness DES regarding clinical outcomes such as all-cause mortality, cardiac mortality, MI, and probable or definite stent thrombosis. Additionally, ultrathin stent DES appears superior to the control group regarding TLF in short-term outcomes extending up to 3 years from PCI.

### Supplementary Information

Supplementary Material 1. Table S1: Search strategy. Table S2: Summary characteristics. Table S3: More details of stent characteristics. Table S4: Sensitivity analysis. Figure S1: Funnel plot of TLF at ≥ 1 year. Figure S2: Funnel plot of TLF at ≥ 2 years. Figure S3: Funnel plot of Cardiac death at ≥ 1 year. Figure S4: Funnel plot of Cardiac death at ≥ 2 years. Figure S5: Funnel plot of Cardiac death at ≥ 3 years. Figure S6: Funnel plot of Target Vessel-Related Myocardial Infarction at ≥ 1 year. Figure S7: Funnel plot of TLR at ≥ 1 year. Figure S8: Funnel plot of TLR at ≥ 2 years. Figure S9: Funnel plot of TLR at ≥ 3 years. Figure S10: Funnel plot of TVR at ≥ 1 year. Figure S11: Funnel plot of TVR at ≥ 2 years. Figure S12: Funnel plot of TVR at ≥ 3 years. Figure S13: Funnel plot of all-cause mortality at ≥ 1 year. Figure S14: Funnel plot of all-cause mortality at ≥ 2 years. Figure S15: Funnel plot of all-cause mortality at ≥ 3 years. Figure S16: Forest plot of patient-oriented composite endpoint. Figure S17: Funnel plot of patient-oriented composite endpoint at ≥ 1 year. Figure S18: Forest plot of any myocardial infarction. Figure S19: Funnel plot of any myocardial infarction at ≥ 1 year. Figure S20: Funnel plot of any myocardial infarction at ≥ 2 years. Figure S21: Funnel plot of any myocardial infarction at ≥ 3 years. Figure S22: Forest plot of any repeat revascularization. Figure S23: Funnel plot of any repeat revascularization at ≥ 1 year. Figure S24: Forest plot of any definite or probable stent thrombosis. Figure S25: Funnel plot of any definite or probable stent thrombosis at ≥ 1 year. Figure S26: Funnel plot of any definite or probable stent thrombosis at ≥ 2 years. Figure S27: Forest plot of definite stent thrombosis. Figure S28: Funnel plot of any definite stent thrombosis at ≥ 1 year. Figure S29: Forest plot of probable stent thrombosis. Figure S30: Forest plot of bleeding. Figure S31A: TLF subgroup analysis at 1 year. Figure S31B: TLF subgroup analysis at 2 year. Figure S31C: TLF subgroup analysis at 3 year. Figure S31D: TLF subgroup analysis at 5 year.

## Data Availability

No datasets were generated or analysed during the current study.

## Abbreviations

ACS: Acute coronary syndrome
CCS: Chronic coronary syndrome
DES: Drug-eluting stents
MI: Myocardial infarction
PCI: Percutaneous coronary intervention
POCE: Patient-oriented composite endpoint
RCTs: Randomized controlled trials
ST: Stent thrombosis
TLF: Target lesion failure
TLR: Target lesion revascularization
TVR: Target vessel revascularization
TVMI: Target vessel myocardial infarction
