# Supplementary material for: Comparative effectiveness of ultrathin vs. standard strut drug-eluting stents: insights from a large-scale meta-analysis with extended follow-up
Source: Eur J Med Res. 2024 Jul 27;29:388. doi: 10.1186/s40001-024-01949-7 (PMC11282633; doi:10.1186/s40001-024-01949-7)
Supplement: Supplementary file 1 — Supplementary Material 1. Table S1: Search strategy. Table S2: Summary characteristics. Table S3: More details of stent characteristics. Table S4: Sensitivity analysis. Figure S1: Funnel plot of TLF at ≥ 1 year. Figure S2: Funnel plot of TLF at ≥ 2 years. Figure S3: Funnel plot of Cardiac death at ≥ 1 year. Figure S4: Funnel plot of Cardiac death at ≥ 2 years. Figure S5: Funnel plot of Cardiac death at ≥ 3 years. Figure S6: Funnel plot of Target Vessel-Related Myocardial Infarction at ≥ 1 year. Figure S7: Funnel plot of TLR at ≥ 1 year. Figure S8: Funnel plot of TLR at ≥ 2 years. Figure S9: Funnel plot of TLR at ≥ 3 years. Figure S10: Funnel plot of TVR at ≥ 1 year. Figure S11: Funnel plot of TVR at ≥ 2 years. Figure S12: Funnel plot of TVR at ≥ 3 years. Figure S13: Funnel plot of all-cause mortality at ≥ 1 year. Figure S14: Funnel plot of all-cause mortality at ≥ 2 years. Figure S15: Funnel plot of all-cause mortality at ≥ 3 years. Figure S16: Forest plot of patient-oriented composite endpoint. Figure S17: Funnel plot of patient-oriented composite endpoint at ≥ 1 year. Figure S18: Forest plot of any myocardial infarction. Figure S19: Funnel plot of any myocardial infarction at ≥ 1 year. Figure S20: Funnel plot of any myocardial infarction at ≥ 2 years. Figure S21: Funnel plot of any myocardial infarction at ≥ 3 years. Figure S22: Forest plot of any repeat revascularization. Figure S23: Funnel plot of any repeat revascularization at ≥ 1 year. Figure S24: Forest plot of any definite or probable stent thrombosis. Figure S25: Funnel plot of any definite or probable stent thrombosis at ≥ 1 year. Figure S26: Funnel plot of any definite or probable stent thrombosis at ≥ 2 years. Figure S27: Forest plot of definite stent thrombosis. Figure S28: Funnel plot of any definite stent thrombosis at ≥ 1 year. Figure S29: Forest plot of probable stent thrombosis. Figure S30: Forest plot of bleeding. Figure S31A: TLF subgroup analysis at 1 year. Figure S31B: TLF subgroup analysis at [file 40001_2024_1949_MOESM1_ESM.docx]

**Supplementary material:**

**Contents:**

**Tables.**Table S1: Search strategy.

Table S2: Summary characteristics

Table S3: More details of stent characteristics

Table S4: Sensitivity analysis.

**Heterogeneity Assessment and Sensitivity Analysis.**

**Figures.**

Figure S1: Funnel plot of TLF at ≥ 1 year.

Figure S2: Funnel plot of TLF at ≥ 2 years.

Figure S3: Funnel plot of Cardiac death at ≥ 1 year.

Figure S4: Funnel plot of Cardiac death at ≥ 2 years.

Figure S5: Funnel plot of Cardiac death at ≥ 3 years.

Figure S6: Funnel plot of Target Vessel-Related Myocardial Infarction at ≥ 1 year.

Figure S7: Funnel plot of TLR at ≥ 1 year.

Figure S8: Funnel plot of TLR at ≥ 2 years.

Figure S9: Funnel plot of TLR at ≥ 3 years.

Figure S10: Funnel plot of TVR at ≥ 1 year.

Figure S11: Funnel plot of TVR at ≥ 2 years.

Figure S12: Funnel plot of TVR at ≥ 3 years.

Figure S13: Funnel plot of all-cause mortality at ≥ 1 year.

Figure S14: Funnel plot of all-cause mortality at ≥ 2 years.

Figure S15: Funnel plot of all-cause mortality at ≥ 3 years.

Figure S16: Forest plot of patient-oriented composite endpoint.

Figure S17: Funnel plot of patient-oriented composite endpoint at ≥ 1 year.

Figure S18: Forest plot of any myocardial infarction.

Figure S19: Funnel plot of any myocardial infarction at ≥ 1 year.

Figure S20: Funnel plot of any myocardial infarction at ≥ 2 years.

Figure S21: Funnel plot of any myocardial infarction at ≥ 3 years.

Figure S22: Forest plot of any repeat revascularization.

Figure S23: Funnel plot of any repeat revascularization at ≥ 1 year

Figure S24: Forest plot of any definite or probable stent thrombosis.

Figure S25: Funnel plot of any definite or probable stent thrombosis at ≥ 1 year

Figure S26: Funnel plot of any definite or probable stent thrombosis at ≥ 2 years.

Figure S27: Forest plot of definite stent thrombosis.

Figure S28: Funnel plot of any definite stent thrombosis at ≥ 1 year

Figure S29: Forest plot of probable stent thrombosis.

Figure S30: Forest plot of bleeding.

Figure S31A: TLF subgroup analysis at 1 year

Figure S31B: TLF subgroup analysis at 2 year

Figure S31C: TLF subgroup analysis at 3 year

Figure S31D: TLF subgroup analysis at 5 year

**Table S1: Search strategy.**

**Details of MeSH search terms were used:** ultrathin, ultrathin strut, biodegradable polymer sirolimus-eluting stents, bioabsorbable stent, thin, thin strut stents, thick stents, normal thickness stents, PCI using ultrathin strut stents, Orsiro stent, Xience stent, BioMime stent, Mistent stent, Supraflex stent, clinical trials, controlled clinical trial, randomized controlled trial, registry, observational studies, human studies, English language

**Time: 20/11/2023**

| Data base | Search terms | Search filed | Search results |
| --- | --- | --- | --- |
| Pubmed | *((Ultrathin strut OR Thin strut OR Orsiro stent) AND (Sirolimus-eluting stent OR SES OR drug eluting stents OR DES) AND (Coronary artery intervention OR Percutaneous coronary intervention OR Coronary angioplasty OR Stent implantation))* | All filed | 434 |
| WOS | *((Ultrathin strut OR Thin strut OR Orsiro stent) AND (Sirolimus-eluting stent OR SES OR drug eluting stents OR DES) AND (Coronary artery intervention OR Percutaneous coronary intervention OR Coronary angioplasty OR Stent implantation))* | All filed | 412 |
| Cochrane  library | *((Ultrathin strut OR Thin strut OR Orsiro stent) AND (Sirolimus-eluting stent OR SES OR drug eluting stents OR DES) AND (Coronary artery intervention OR Percutaneous coronary intervention OR Coronary angioplasty OR Stent implantation))* | All filed | 240 |
| Scopus | *((Ultrathin strut OR Thin strut OR Orsiro stent) AND (Sirolimus-eluting stent OR SES OR drug eluting stents OR DES) AND (Coronary artery intervention OR Percutaneous coronary intervention OR Coronary angioplasty OR Stent implantation))* | All filed | 578 |

Table S2: Summary characteristics

| Study ID | Study design | country | Center | Recruitment | Number of patients | | Follow up | primary outcome | secondary outcome |
| --- | --- | --- | --- | --- | --- | --- | --- | --- | --- |
|  |  |  |  |  |  |  |  |  |  |
|  |  |  |  |  | **I** | **C** |  |  |  |
| DESSOLVE-C[^(20)^](https://sciwheel.com/work/citation?ids=15808263&pre=&suf=&sa=0&dbf=0) | RCT | China | 16 Center | July 2015 to November 2017 | 216 | 212 | 1-Year | - | - |
| CASTLE[^(21)^](https://sciwheel.com/work/citation?ids=15808276&pre=&suf=&sa=0&dbf=0) | RCT | Japan | 65 Center | May 2019 to March 2020 | 722 | 718 | 1-Year | TLF at 1 year, defined as the composite of cardiac death, TVMI, and TLR | POCE of all-cause death, all MI, and all revascularization, and ST, major adverse cardiac events. |
| SCAAR[^(22)^](https://sciwheel.com/work/citation?ids=11345518&pre=&suf=&sa=0&dbf=0) | prospective registry | Sweden | 30 hospitals | October 2011 to June 2017 | 4561 | 69570 | 1 and 2 Year | Principle outcomes: ST, clinically relevant restenosis, TLR by PCI, MI and all-Cause Mortality. | |
| BIODEGRADE[^(23,24)^](https://sciwheel.com/work/citation?ids=12023987,15808275&pre=&pre=&suf=&suf=&sa=0,0&dbf=0&dbf=0) | RCT | South Korea | 25 Centers | July 2014 to September 2017 | 1167 | 1160 | 18 months and 3 Year | Target lesion failure . | Death, Target vessel-related myocardial infarction. |
| SORT OUT IX[^(25,26)^](https://sciwheel.com/work/citation?ids=9511697,15808274&pre=&pre=&suf=&suf=&sa=0,0&dbf=0&dbf=0) | RCT | Denmark | 3 centers | Dec 14, 2015 to April 21, 2017 | 1,579 | 1,572 | 1 and 2 Year | TLF | Cardiac death, MI and definite ST |
| BIOFLOW VI[^(27)^](https://sciwheel.com/work/citation?ids=9511693&pre=&suf=&sa=0&dbf=0) | RCT | China | 11 center | July 2014 to September 2016 | 220 | 220 | 1 Year | All-cause death, all MI, and TLR; TLF, cardiac death, TVMI, and TLR; target vessel failure, TVR, and definite or probable ST | |
| BIOSTEMI[^(28–30)^](https://sciwheel.com/work/citation?ids=9511654,10675570,15808272&pre=&pre=&pre=&suf=&suf=&suf=&sa=0,0,0&dbf=0&dbf=0&dbf=0) | RCT | Switzerland | 10 centers | April 26, 2016, to Mar 9, 2018 | 649 | 651 | 1, 2, and 5 Year | TLF, a composite of cardiac death, TVMI, and clinically indicated TLR as assessed at 12 months. | - |
| BIOFLOW-IV[^(31,32)^](https://sciwheel.com/work/citation?ids=9511651,15808270&pre=&pre=&suf=&suf=&sa=0,0&dbf=0&dbf=0) | RCT | Japan, Europe, Australia and Israel | 46 center | Sept 2013 to Jan 2015 | 385 | 190 | 1 and 5 Year | TVF | TLF, TLR, TVR,MI and Death |
| TALENT[^(33–35)^](https://sciwheel.com/work/citation?ids=9511707,15808269,15808268&pre=&pre=&pre=&suf=&suf=&suf=&sa=0,0,0&dbf=0&dbf=0&dbf=0) | RCT | Netherlands, Poland, the UK, Spain, Bulgaria, Hungary, and Italy | 23 Center | October 2016 to July 2017 | 720 | 715 | 1, 2, and 3 Year | Non-inferiority comparison at 12 months between the Supraflex group and the Xience group regarding a device-oriented composite endpoint of cardiac death, TVMI, and TLR | All-cause death, MI, revascularization, TVF, cardiac death, TVMI, TVR, POCE, and ST |
| MeriT-V[^(36,37)^](https://sciwheel.com/work/citation?ids=6044144,15808267&pre=&pre=&suf=&suf=&sa=0,0&dbf=0&dbf=0) | RCT | Europe and Brazil | 15 center | Nov2014 to December 2016 | 170 | 86 | 1 and 2 Year | Three-point MACEs inclusive of cardiac death, TVR, and all MI was considered for the combined safety and efficacy evaluation at the 2-year landmark. | Individually evaluated, including TLR and late ST for the population that completed the 2-year follow-up. |
| BIONYX 3-year [^(38–40)^](https://sciwheel.com/work/citation?ids=6044080,9511691,14142755&pre=&pre=&pre=&suf=&suf=&suf=&sa=0,0,0&dbf=0&dbf=0&dbf=0) | RCT | Netherlands, Belgium, and Israel | 7 Centers | Oct 7, 2015 to Dec 23, 2016 | 1,245 | 1,243 | 1,2, and 3 Year | TVF, a composite of cardiac death, TVMI, or TVR | All-cause death; MI, TLR, major bleeding; and ST |
| DESSOLVE III[^(41–43)^](https://sciwheel.com/work/citation?ids=6044054,8657768,9114680&pre=&pre=&pre=&suf=&suf=&suf=&sa=0,0,0&dbf=0&dbf=0&dbf=0) | RCT | Germany, France, Netherlands, and Poland. | 20 Center | March 20 to Dec 3, 2015 | 703 | 695 | 1, 2, and 3 Year | Device (DOCE); cardiac death, TVMI, or TLR | POCE, defined as the composite of any death, MI, or revascularization. |
| CARDIOBASE Bern PCI Registry[^(44)^](https://sciwheel.com/work/citation?ids=6044089&pre=&suf=&sa=0&dbf=0) | prospective registry | Switzerland | Bern University Hospital | March 2011 to June 2015 | 1451 | 1451 | 1 Year | DOCE, including cardiac death, TVMI, and TLR | - |
| ORIENT[^(45,46)^](https://sciwheel.com/work/citation?ids=6042386,8657763&pre=&pre=&suf=&suf=&sa=0,0&dbf=0&dbf=0) | RCT | South Korea | 8 Centers | October 2013 to June 2014 | 250 | 122 | 1,2, and 3 Year | All-cause and cardiac death, TLR, TVR, MI (target or non-target vessel-related), definite or probable ST, TLF, a composite of cardiac death, TLR and TVMI at 12 months | |
| PRISON IV[^(47–49)^](https://sciwheel.com/work/citation?ids=5174735,11428626,15808265&pre=&pre=&pre=&suf=&suf=&suf=&sa=0,0,0&dbf=0&dbf=0&dbf=0) | RCT | Netherlands and Belgium | 2 Belgian and 6 Dutch high-volume PCI centers. | February 2012 to June 2015 | 165 | 165 | 1, 3, and 5 Year | Segment late lumen loss on 9-month angiography assessment. | Secondary angiographic endpoints included in-stent late lumen loss, MLD, in-stent and in-segment percentage of diameter stenosis, binary restenosis, and reocclusions at 9 months. |
| BIO-RESORT[^(50–53)^](https://sciwheel.com/work/citation?ids=4959871,6042352,7392883,14539935&pre=&pre=&pre=&pre=&suf=&suf=&suf=&suf=&sa=0,0,0,0&dbf=0&dbf=0&dbf=0&dbf=0) | RCT | Netherlands | 4 Center | Dec 21, 2012, to Aug 24, 2015 | 1169 | 2345 | 1, 2, 3, and 5 Year | The primary composite endpoint of TVF assessed device efficacy and patient safety and consisted of cardiac death, TVMI, or TVR. | Secondary endpoints included: TLR, TLF, a composite of cardiac death, MI not clearly attributable to a non-target vessel, or TLR, MACE, a composite of all-cause death, MI, or emergent coronary bypass surgery, or repeat TLR, POCE, a composite of all-cause death, MI, or repeat revascularization, and definite-or-probable ST. |
| SORT OUT VII[^(54–57)^](https://sciwheel.com/work/citation?ids=4960214,6045740,9554577,15808264&pre=&pre=&pre=&pre=&suf=&suf=&suf=&suf=&sa=0,0,0,0&dbf=0&dbf=0&dbf=0&dbf=0) | RCT | Denmark | Multi center | November 2012 to February 2014 | 1261 | 1264 | 1,2,3,4, and 5 Year | TLF; Five-year TLF did not differ between O-SES (12.4%) and N-BES (13.1%). | Cardiac death |
| BIOFLOW V[^(58–61)^](https://sciwheel.com/work/citation?ids=6044037,6044079,9532554,13636202&pre=&pre=&pre=&pre=&suf=&suf=&suf=&suf=&sa=0,0,0,0&dbf=0&dbf=0&dbf=0&dbf=0) | RCT | Asia, Europe, Israel, and North America | 90 hospitals | May 8, 2015 to March 31, 2016 | 884 | 450 | 1,2,3, and 5 Year | 5-year primary endpoint assessment consisted of TLF, cardiac death, TVMI, or TLR and MACE (all-cause death, MI, or TLR), TVF (cardiac death, TVMI, or TVR). Also, individual components of the composite endpoints, and definite or probable ST Periprocedural MI was defined |  |
| DESSOLVE I and II[^(62–64)^](https://sciwheel.com/work/citation?ids=2797821,4960817,8657771&pre=&pre=&pre=&suf=&suf=&suf=&sa=0,0,0&dbf=0&dbf=0&dbf=0) | RCT | New Zealand, and Europe | Multi-centers | November 2010 to March 2011 | 123 | 61 | 2 and 5 Year | MACE was a composite of any death, MI, TVR. TLF was defined as cardiac death, MI, or TLR. TVF | All-cause mortality, rates of Q-wave and non-Q-wave MI, rates or TLR and TVR, rates of TLF and TVF |
| BIOFLOW-II[^(65,66)^](https://sciwheel.com/work/citation?ids=2797831,6045856&pre=&pre=&suf=&suf=&sa=0,0&dbf=0&dbf=0) | RCT | 8 European countries | 24 centers | July 2011 and March 2012 | 298 | 154 | 1 and 5 Year | In-stent late lumen loss | TLF, a composite of cardiac death, TVMI, and TLR; TVF TVR, all- cause mortality,MI; and definite ST |
| BIOSCIENCE[^(67–69)^](https://sciwheel.com/work/citation?ids=2382666,6044334,8657748&pre=&pre=&pre=&suf=&suf=&suf=&sa=0,0,0&dbf=0&dbf=0&dbf=0) | RCT | Switzerland | 9 center | February 2012 to May 2013 | 1063 | 1056 | 1, 2, and 5 Year | TLF, was a composite of cardiac death, TVMI, and TLR within 12 months. | - |

Intervention (I), Control (C).

**Abbreviations:** Myocardial infarction (MI), Patient-oriented composite endpoint (POCE), Stent thrombosis (ST), Target lesion failure (TLF), Target lesion revascularization (TLR), Target vessel revascularization (TVR), Target vessel myocardial infarction (TVMI)

**Table S3: Sensitivity analysis.**

| Outcome | No. of  Participants (/) | No. of  trials | Quantitative data synthesis | | | | Heterogeneity analysis | | |
| --- | --- | --- | --- | --- | --- | --- | --- | --- | --- |
|  |  |  | MD | 95% CI | Z value | p-value | df | p-value | I2 (%) |
| Any repeat revascularization ≥ 3 years. | | | | | | | | | |
| BIODEGRADE | 4083/5113 | 5 | 0.97 | [0.82,1.14] | 0.42 | 0.67 | 4 | 0.14 | 41% |
| BIONYX | 4005/5030 | 5 | 0.90 | [0.74,1.10] | 1.03 | 0.31 | 4 | 0.08 | 51% |
| BIO-RESORT | 4081/3928 | 5 | 0.93 | [0.76,1.15] | 0.64 | 0.52 | 4 | 0.05 | 58% |
| DESSOLVE III | 4547/5580 | 5 | 0.97 | [0.82,1.15] | 0.33 | 0.74 | 4 | 0.17 | 38% |
| ORIENT | 5004/6156 | 5 | 0.91 | [0.77,1.06] | 1.21 | 0.23 | 4 | 0.10 | 48% |
| TALENT | 4530/5558 | 5 | 0.89 | [0.75,1.05] | 1.00 | 0.15 | 4 | 0.15 | 41% |
| Patient-oriented composite end point ≥ 3 years. | | | | | | | | | |
| BIODEGRADE | 4488/5329 | 6 | 1.04 | [0.93,1.16] | 0.67 | 0.50 | 5 | 0.20 | 32% |
| BIOFLOW-IV | 5270/6299 | 6 | 1.00 | [0.87,1.14] | 0.06 | 0.95 | 5 | 0.04 | 58% |
| BIO-RESORT | 4486/4144 | 6 | 1.01 | [0.87,1.18] | 0.19 | 0.85 | 5 | 0.05 | 55% |
| DESSOLVE III | 4952/5796 | 6 | 1.02 | [0.89,1.18] | 0.33 | 0.74 | 5 | 0.07 | 50% |
| ORIENT | 5405/6367 | 6 | 0.99 | [0.87,1.12] | 0.20 | 0.84 | 5 | 0.06 | 54% |
| SORT OUT VII | 4394/5225 | 6 | 0.95 | [0.85,1.05] | 1.00 | 0.32 | 5 | 0.33 | 13% |
| TALENT | 4935/5774 | 6 | 0.99 | [0.85,1.14] | 0.20 | 0.84 | 5 | 0.04 | 57% |
| Target lesion revascularization ≥ 1 year. | | | | | | | | | |
| BIODEGRADE | 17545/82688 | 19 | 0.80 | [0.66,0.98] | 2.14 | 0.03 | 18 | 0.04 | 39% |
| BIOFLOW-II | 18414/83694 | 19 | 10.79 | [0.65,0.97] | 2.25 | 0.02 | 18 | 0.04 | 40% |
| BIOFLOW-IV | 18340/83667 | 19 | 0.78 | [0.64,0.95] | 2.52 | 0.01 | 18 | 0.05 | 37% |
| BIOFLOW V | 17880/83426 | 19 | 0.79 | [0.64,0.96] | 2.31 | 0.02 | 18 | 0.04 | 40% |
| BIOFLOW-VI | 18492/83628 | 19 | 0.79 | [0.65,0.96] | 2.32 | 0.02 | 18 | 0.04 | 40% |
| BIONYX | 17467/82605 | 19 | 0.79 | [0.64,0.98] | 2.20 | 0.03 | 18 | 0.04 | 40% |
| BIO-RESORT | 17543/81503 | 19 | 0.77 | [0.63,0.95] | 2.49 | 0.01 | 18 | 0.05 | 38% |
| BIOSCIENCE | 17649/82792 | 19 | 0.75 | [0.62,0.91] | 2.94 | 0.003 | 18 | 0.12 | 29% |
| BIOSTEMI | 18063/83197 | 19 | 0.80 | [0.66,0.98] | 2.14 | 0.03 | 18 | 0.04 | 39% |
| Buccheri et al. 2021 (SCAAR) | 14151/14278 | 19 | 0.81 | [0.66,1.00] | 1.95 | 0.05 | 18 | 0.06 | 36% |
| CASTLE | 17990/83130 | 19 | 0.80 | [0.65,0.97] | 2.22 | 0.03 | 18 | 0.04 | 40% |
| DESSOLVE II | 18595/83788 | 19 | 0.79 | [0.65,0.97] | 2.30 | 0.02 | 18 | 0.04 | 40% |
| DESSOLVE III | 18009/83153 | 19 | 0.79 | [0.64,0.97] | 2.24 | 0.03 | 18 | 0.04 | 40% |
| meriT-V | 18544/83764 | 19 | 0.79 | [0.65,0.96] | 2.36 | 0.02 | 18 | 0.04 | 40% |
| ORIENT | 18462/83726 | 19 | 0.80 | [0.65,0.97] | 2.27 | 0.02 | 18 | 0.04 | 40% |
| PRISON IV | 18547/83683 | 19 | 0.76 | [0.64,0.90] | 3.19 | 0.001 | 18 | 0.19 | 22% |
| SORT OUT IX | 17133/82276 | 19 | 0.84 | [0.71,0.98] | 2.15 | 0.03 | 18 | 0.29 | 13% |
| SORT OUT VII | 17451/82584 | 19 | 0.80 | [0.65,0.99] | 2.10 | 0.04 | 18 | 0.04 | 40% |
| TALENT | 17992/83133 | 19 | 0.79 | [0.64,0.97] | 2.25 | 0.02 | 18 | 0.04 | 40% |
| Yamaji et al. 2018 (CARDIOBASE) | 17261/82397 | 19 | 0.76 | [0.63,0.93] | 2.64 | 0.008 | 18 | 0.07 | 34% |
| Target lesion revascularization ≥ 2 years. | | | | | | | | | |
| BIOFLOW-IV | 14304/79784 | 13 | 0.77 | [0.66,0.90] | 3.27 | 0.001 | 12 | 0.13 | 31% |
| BIOFLOW V | 13873/79567 | 13 | 0.81 | [0.68,0.97] | 2.28 | 0.02 | 12 | 0.05 | 43% |
| BIONYX | 13444/78731 | 13 | 0.79 | [0.65,0.95] | 2.44 | 0.01 | 12 | 0.03 | 46% |
| BIO-RESORT | 13520/77629 | 13 | 0.79 | [0.65,0.96] | 2.42 | 0.02 | 12 | 0.03 | 47% |
| BIOSCIENCE | 13626/78918 | 13 | 0.76 | [0.64,0.90] | 3.14 | 0.002 | 12 | 0.11 | 34% |
| BIOSTEMI | 14040/79323 | 13 | 0.81 | [0.68,0.97] | 2.26 | 0.02 | 12 | 0.05 | 42% |
| Buccheri et al. 2021 (SCAAR) | 10128/10404 | 13 | 0.81 | [0.67,0.99] | 2.05 | 0.04 | 12 | 0.05 | 43% |
| DESSOLVE II | 14569/79914 | 13 | 0.79 | [0.66,0.95] | 2.56 | 0.01 | 12 | 0.03 | 47% |
| DESSOLVE III | 13986/79279 | 13 | 0.79 | [0.65,0.95] | 2.44 | 0.01 | 12 | 0.03 | 47% |
| meriT-V | 14521/79890 | 13 | 0.78 | [0.66,0.93] | 2.77 | 0.006 | 12 | 0.05 | 43% |
| ORIENT | 14439/79852 | 13 | 0.80 | [0.66,0.96] | 2.45 | 0.01 | 12 | 0.03 | 47% |
| SORT OUT IX | 13110/78402 | 13 | 0.83 | [0.70,0.98] | 2.22 | 0.03 | 12 | 0.15 | 30% |
| SORT OUT VII | 13428/78710 | 13 | 0.79 | [0.65,0.96] | 2.35 | 0.02 | 12 | 0.03 | 47% |
| TALENT | 13969/79259 | 13 | 0.77 | [0.64,0.92] | 2.87 | 0.004 | 12 | 0.06 | 41% |
| Target lesion revascularization ≥ 3 years. | | | | | | | | | |
| BIODEGRADE | 6782/7187 | 9 | 0.95 | [0.73,1.22] | 0.42 | 0.67 | 8 | 0.01 | 58% |
| BIOFLOW-IV | 7564/8157 | 9 | 0.85 | [0.68,1.05] | 1.49 | 0.14 | 8 | 0.05 | 48% |
| BIOFLOW V | 7065/7897 | 9 | 0.96 | [0.75,1.22] | 0.37 | 0.71 | 8 | 0.03 | 53% |
| BIONYX | 6704/7104 | 9 | 0.90 | [0.67,1.20] | 0.72 | 0.47 | 8 | 0.005 | 63% |
| BIO-RESORT | 6780/6002 | 9 | 0.92 | [0.69,1.23] | 0.20 | 0.58 | 8 | 0.005 | 64% |
| DESSOLVE III | 7246/7654 | 9 | 0.92 | [0.69,1.23] | 0.58 | 0.56 | 8 | 0.005 | 64% |
| ORIENT | 7699/8225 | 9 | 0.91 | [0.70,1.19] | 0.69 | 0.49 | 8 | 0.005 | 64% |
| PRISON IV | 7784/8182 | 9 | 0.83 | [0.67,1.04] | 1.63 | 0.10 | 8 | 0.06 | 46% |
| SORT OUT VII | 6688/7083 | 9 | 0.91 | [0.68,1.23] | 0.61 | 0.54 | 8 | 0.005 | 64% |
| TALENT | 7229/7632 | 9 | 0.89 | [0.67,1.19] | 0.77 | 0.44 | 8 | 0.006 | 63% |

MD: mean difference; CI: confidence interval; df: degrees of freedom; RR: risk ratio

**Heterogeneity Assessment and Sensitivity Analysis.**

**Primary Outcome**

**Target Lesion Failure (TLF)**

The pooled studies were homogenous at ≥ 1 year (I^2^= 0%, P= 0.94), at ≥ two years (I^2^= 0%, P= 0.65), at ≥ three years (I^2^= 35%, P= 0.15), and at 5 years (I^2^= 0%, P= 0.54). Regarding TLF at ≥ 1 year and ≥ 2 years, we did not find asymmetry in the funnel plot, meaning that there was no significant publication bias (Egger’s P-value = 0.63) **(Figure S1)** and (Egger’s P-value = 0.44) **(Figure S2)**, respectively.

**Cardiac Death**

The pooled studies were homogenous at ≥ 1 year (I^2^= 0%, P= 0.61), at ≥ 2 years (I^2^= 0%, P= 0.94), at ≥ 3 years (I^2^= 0%, P= 0.77), and at 5 years (I^2^= 0%, P= 0.99). Regarding cardiac death at ≥ 1 year, at ≥ 2 years, and at ≥ 3 years, no asymmetry was observed in the funnel plot, indicating no significant publication bias (Egger’s P-value = 0.63) **(Figure S3)**, (Egger’s P-value = 0.55) **(Figure S4)**, and (Egger’s P-value = 0.47) **(Figure S5)**, respectively.

**Target Vessel-Related Myocardial Infarction (TVMI)**

The pooled studies were homogenous at ≥ 1 year (I^2^= 0%, P= 0.86), at ≥ 2 years (I^2^= 0%, P= 0.72), at ≥ 3 years (I^2^= 32%, P= 0.18), and at 5 years (I^2^= 33%, P= 0.16). Regarding TVMI at ≥ 1 year, we did not find asymmetry in the funnel plot, meaning that there was no significant publication bias (Egger’s P-value = 0.33) **(Figure S6)**.

**Target Lesion Revascularization (TLR)**

The pooled studies were homogenous at five years (I^2^= 26%, P= 0.21). However, pooled studies were heterogeneous regarding TLR at ≥ 1 year (I^2^= 37%, P= 0.05), at ≥ 2 years (I^2^= 43%, P= 0.05), and at ≥3 years (I^2^= 59%, P= 0.009). Regarding TLR at ≥ 1 year, the heterogeneity was best resolved by excluding BIOSCIENCE (I^2^= 29%, P= 0.12), PRISON IV (I^2^= 22%, P= 0.19), and SORT OUT IX (I^2^= 13%, P= 0.29). Regarding TLR at ≥2 years, the heterogeneity was best resolved by excluding BIOFLOW-IV (I^2^= 31%, P= 0.13), BIOSCIENCE (I^2^= 34%, P= 0.11), and SORT OUT IX (I^2^= 30%, P= 0.15). Regarding TLR at ≥ 3 years, the heterogeneity was not resolved by leave-one-out sensitivity analysis **(Table S2)**. Regarding TLR at ≥1 year, at ≥ 2 years, and at ≥ 3 years, we did not find asymmetry by inspecting the funnel plot, meaning that there was no significant publication bias (Egger’s P-value = 0.67) **(Figure S7)**, (Egger’s P-value = 0.19) **(Figure S8)**, and (Egger’s P-value = 0.12) **(Figure S9)**, respectively.

**Secondary outcome**

**Target Vessel Revascularization (TVR)**

The pooled studies were homogenous at ≥ 1 year (I^2^= 0%, P= 0.60), at ≥ 2 years (I^2^= 5%, P= 0.39), at ≥ 3 years (I^2^= 1%, P= 0.43), and at 5 years (I^2^= 27%, P= 0.21). Regarding TVR at ≥ 1 year and at ≥ 3 years, we did not find asymmetry in the funnel plot, meaning that there was no significant publication bias (Egger’s P-value = 0.11) **(Figure S10)** and (Egger’s P-value = 0.58) **(Figure S11)**, respectively. Regarding TVR at ≥2 years, we found asymmetry in the funnel plot with significant publication bias (Egger’s P-value = 0.09) **(Figure S12)**.

**All-Cause Mortality**

The pooled studies were homogenous at ≥ 1 year (I^2^= 3%, P= 0.42), at ≥ two years (I^2^= 0%, P= 0.68), at ≥ 3 years (I^2^= 7%, P= 0.38), and at 5 years (I^2^= 29%, P= 0.19). Regarding all-cause mortality at ≥ 1 year and at ≥ 2 years, we did not find asymmetry by inspecting the funnel plot, meaning that there was no significant publication bias (Egger’s P-value = 0.50) **(Figure S13)** and (Egger’s P-value = 0.29) **(Figure S14)**, respectively. Regarding all-cause mortality at ≥ 3 years, we found asymmetry by inspecting the funnel plot with significant publication bias (Egger’s P-value = 0.09) **(Figure S15)**.

**Patient oriented composite endpoint (POCE)**

The pooled studies were homogenous at ≥ 1 year (I^2^= 9%, P= 0.36), at ≥ 2 years (I^2^= 0%, P= 0.64), and at 5 years (I^2^= 0%, P= 0.80). However, pooled studies were heterogeneous regarding POCE at ≥ 3 years (I^2^= 50%, P= 0.06). The heterogeneity was best resolved by excluding BIODEGRADE and SORT OUT VII (I^2^= 32%, P= 0.20) and (I^2^= 13%, P= 0.33), respectively **(Table S2)**. Regarding POCE at ≥ 1 year, we did not find asymmetry in the funnel plot, meaning that there was no significant publication bias (Egger’s P value = 0.68) **(Figure S17)**.

**Myocardial Infarction (MI)**

The pooled studies were homogenous at ≥ 1 year (I^2^= 17%, P= 0.24), at ≥ 2 years (I^2^= 0%, P= 0.58), at ≥ 3 years (I^2^= 24%, P= 0.22), and at 5 years (I^2^= 8%, P= 0.37). Regarding MI at ≥ 1 year, at ≥ 2 years, and at ≥ 3 years, we did not find asymmetry in the funnel plot, meaning that there was no significant publication bias (Egger’s P-value = 0.37) **(Figure S19)**, (Egger’s P-value = 0.12) **(Figure S20)**, and (Egger’s P-value = 0.87) **(Figure S21)**, respectively.

**Repeat Revascularization**

The pooled studies were homogenous at ≥ 1 year (I^2^= 1%, P= 0.43), at ≥2 years (I^2^= 39%, P= 0.13), and at 5 years (I^2^= 24%, P= 0.27). However, pooled studies were heterogeneous regarding repeat revascularization at ≥ 3 years (I^2^= 47%, P= 0.09). The heterogeneity was best resolved by excluding BIODEGRADE (I^2^= 41%, P= 0.14), DESSOLVE III (I^2^= 38%, P= 0.17), ORIENT (I^2^= 48%, P= 0.10), and TALENT (I^2^= 41%, P= 0.15) **(Table S2)**. Regarding repeat revascularization at ≥ 1 year, we did not find asymmetry by inspecting the funnel plot, meaning that there was no significant publication bias (Egger’s P-value = 0.21) **(Figure S23)**.

**Definite or Probable Stent Thrombosis (ST)**

The pooled studies were homogenous at ≥ 1 year (I^2^= 0%, P= 0.76), at ≥ two years (I^2^= 0%, P= 0.55), at ≥ 3 years (I^2^= 13%, P= 0.33), and at 5 years (I^2^= 28%, P= 0.20). Regarding definite or probable ST at ≥ 1 year and at ≥ 2 years, we did not find asymmetry in the funnel plot, meaning that there was no significant publication bias (Egger’s P-value = 0.31) **(Figure S25)** and (Egger’s P-value = 0.89) **(Figure S26)**, respectively.

**Definite Stent Thrombosis (ST)**

The pooled studies were homogenous at ≥ 1 year (I^2^= 21%, P= 0.25), at ≥ 2 years (I^2^= 4%, P= 0.40), at ≥ 3 years (I^2^= 37%, P= 0.16), and at 5 years (I^2^= 10%, P= 0.35). Regarding definite ST at ≥ 1 year, we did not find asymmetry in the funnel plot, meaning that there was no significant publication bias (Egger’s P-value = 0.70) **(Figure S28)**.

**Probable Stent Thrombosis (ST)**

The pooled studies were homogenous at ≥ 1 year (I^2^= 0%, P= 0.82), at ≥ 2 years (I^2^= 0%, P= 0.50), at ≥ 3 years (I^2^= 0%, P= 0.81), and at 5 years (I^2^= 0%, P= 0.86).

**Bleeding**

The pooled studies were homogenous at ≥ 1 year (I^2^= 0%, P= 0.71), at ≥ 2 years (I^2^= 0%, P= 0.54), at ≥ 3 years (I^2^= 0%, P= 0.33), and at 5 years (I^2^= 0%, P= 0.92).


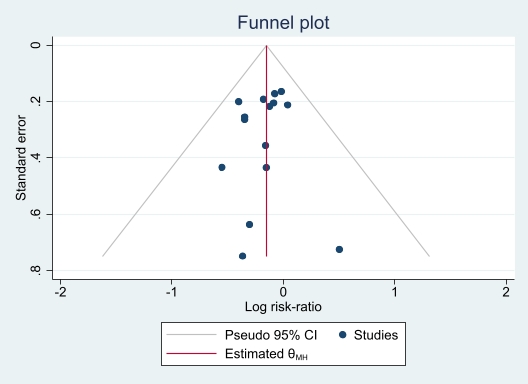


**Figure S1: Funnel plot of TLF at ≥ 1 year.**


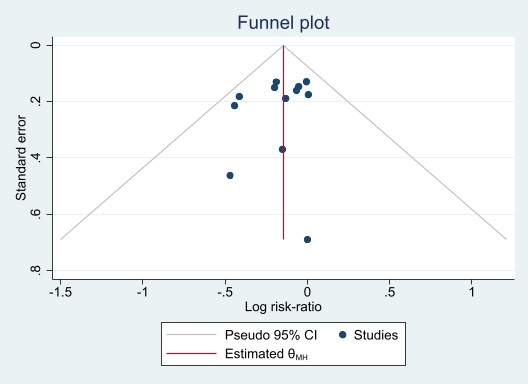


**Figure S2: Funnel plot of TLF at ≥ 2 years.**


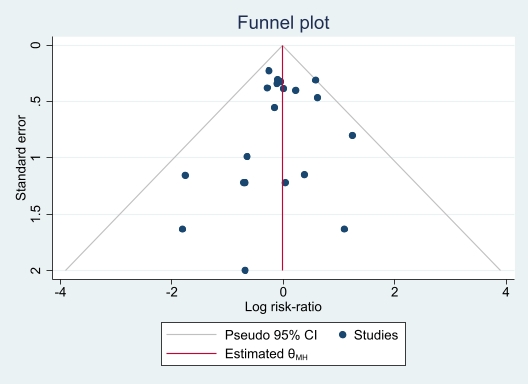


**Figure S3: Funnel plot of Cardiac death at ≥ 1 year.**


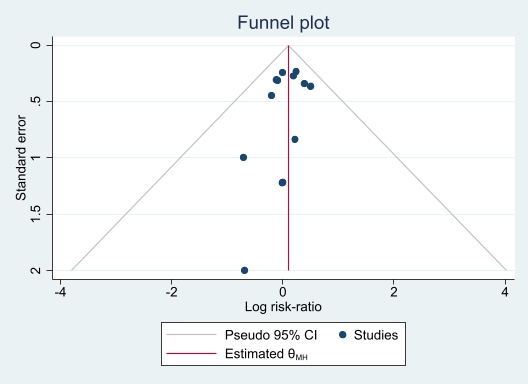


**Figure S4: Funnel plot of Cardiac death at ≥ 2 years.**


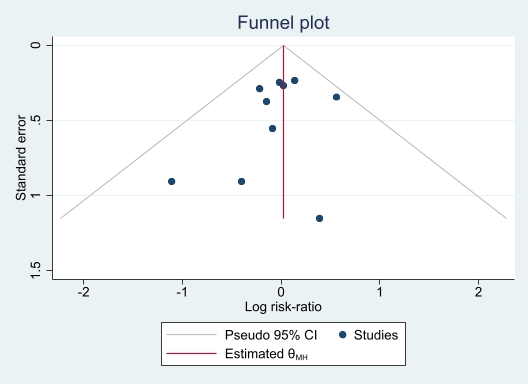


**Figure S5: Funnel plot of Cardiac death at ≥ 3 years.**


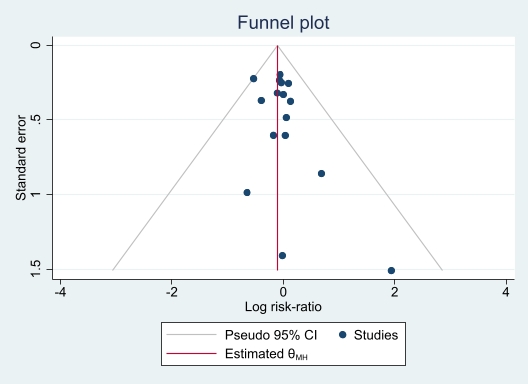


**Figure S6: Funnel plot of Target Vessel-Related Myocardial Infarction at ≥ 1 year.**


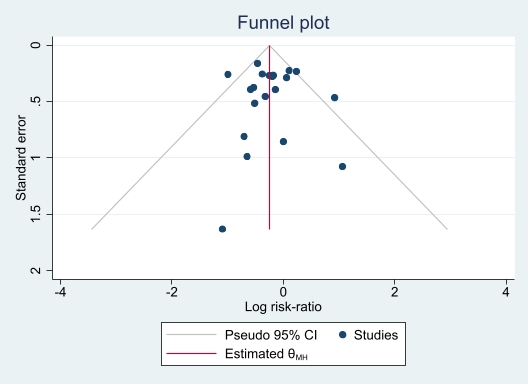


**Figure S7: Funnel plot of TLR at ≥ 1 year.**


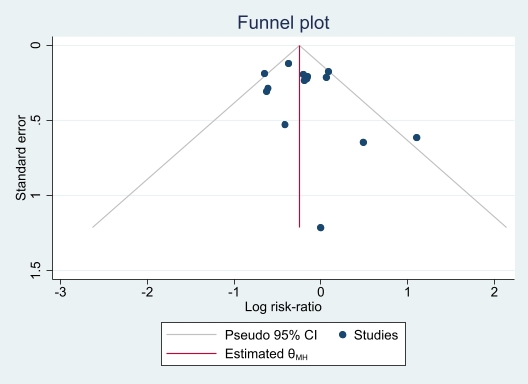


**Figure S8: Funnel plot of TLR at ≥ 2 years.**


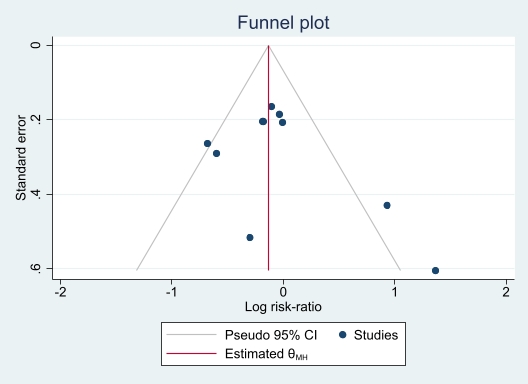


**Figure S9: Funnel plot of TLR at ≥ 3 years.**


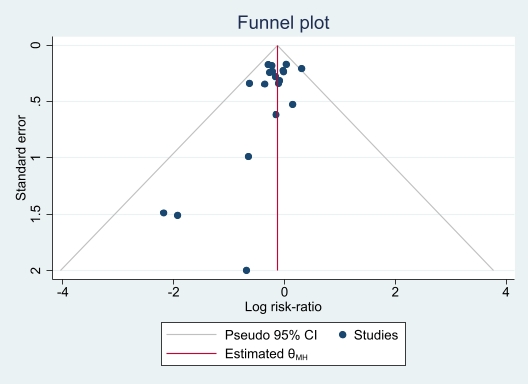


**Figure S10: Funnel plot of TVR at ≥ 1 year.**


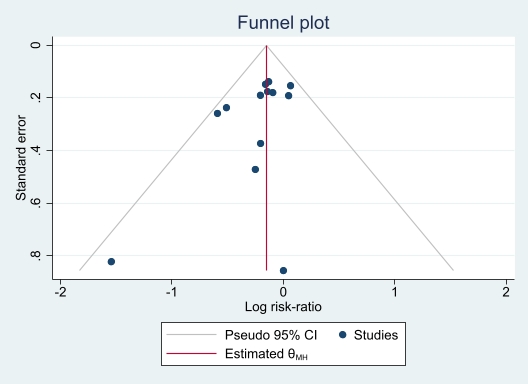


**Figure S11: Funnel plot of TVR at ≥ 2 years.**


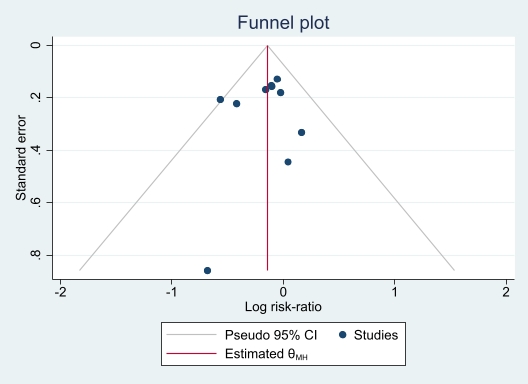


**Figure S12: Funnel plot of TVR at ≥ 3 years.**


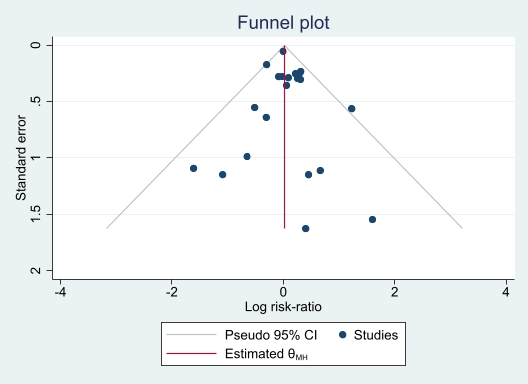


**Figure S13: Funnel plot of all-cause mortality at ≥ 1 year.**


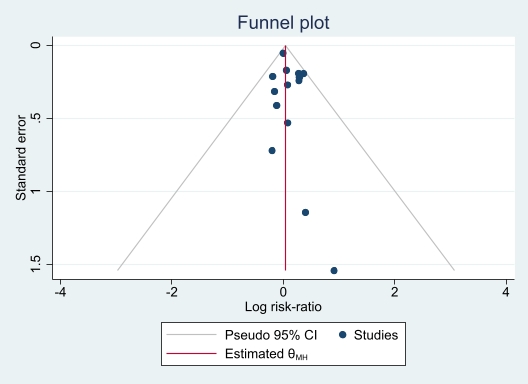


**Figure S14: Funnel plot of all-cause mortality at ≥ 2 years.**


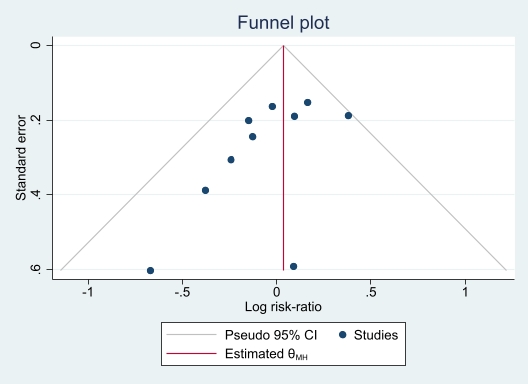


**Figure S15: Funnel plot of all-cause mortality at ≥ 3 years.**


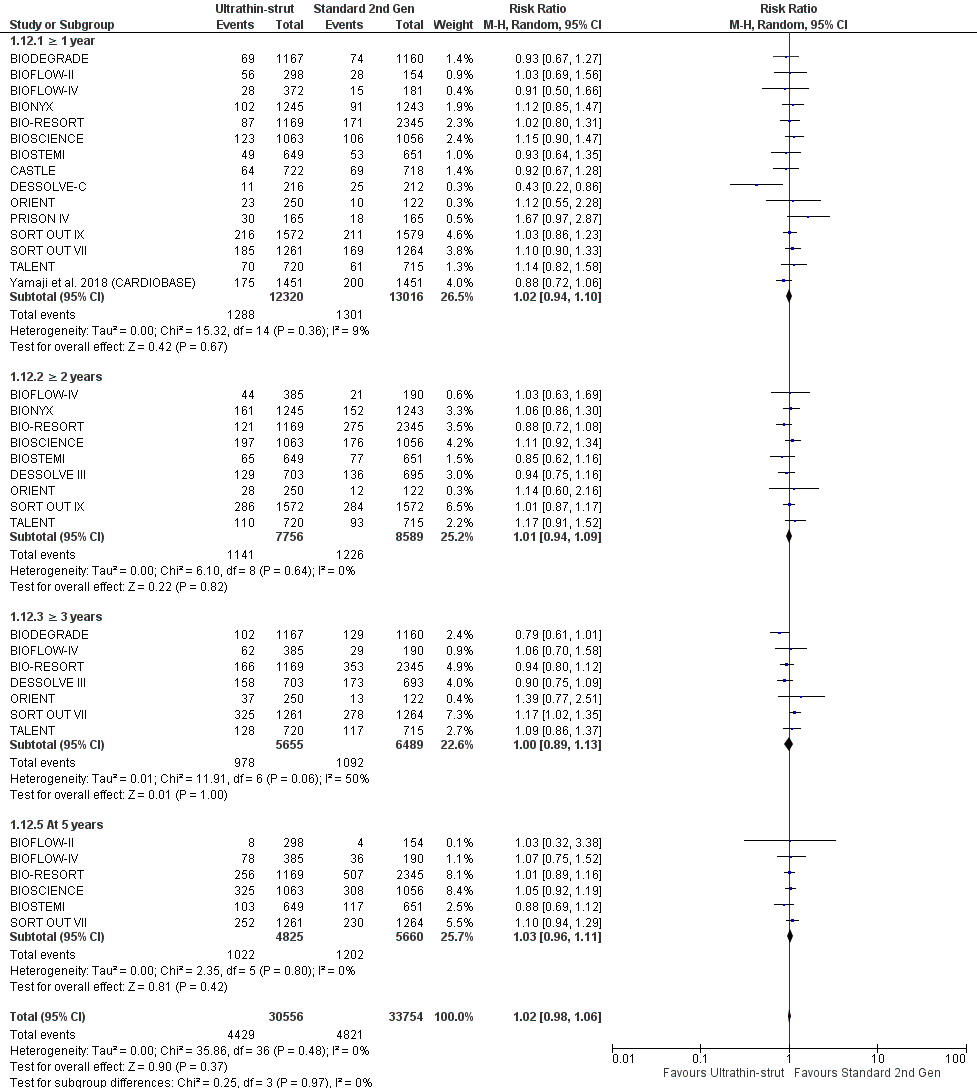


**Figure S16: Forest plot of patient-oriented composite endpoint.**


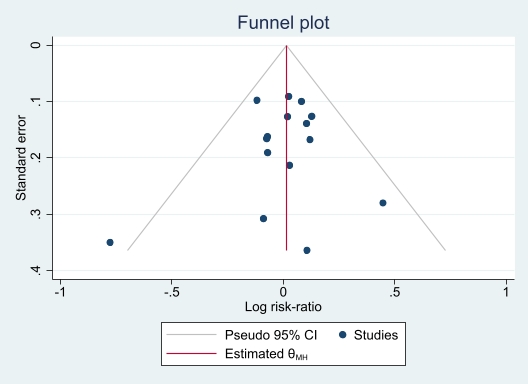


**Figure S17: Funnel plot of patient-oriented composite endpoint at ≥ 1 year.**


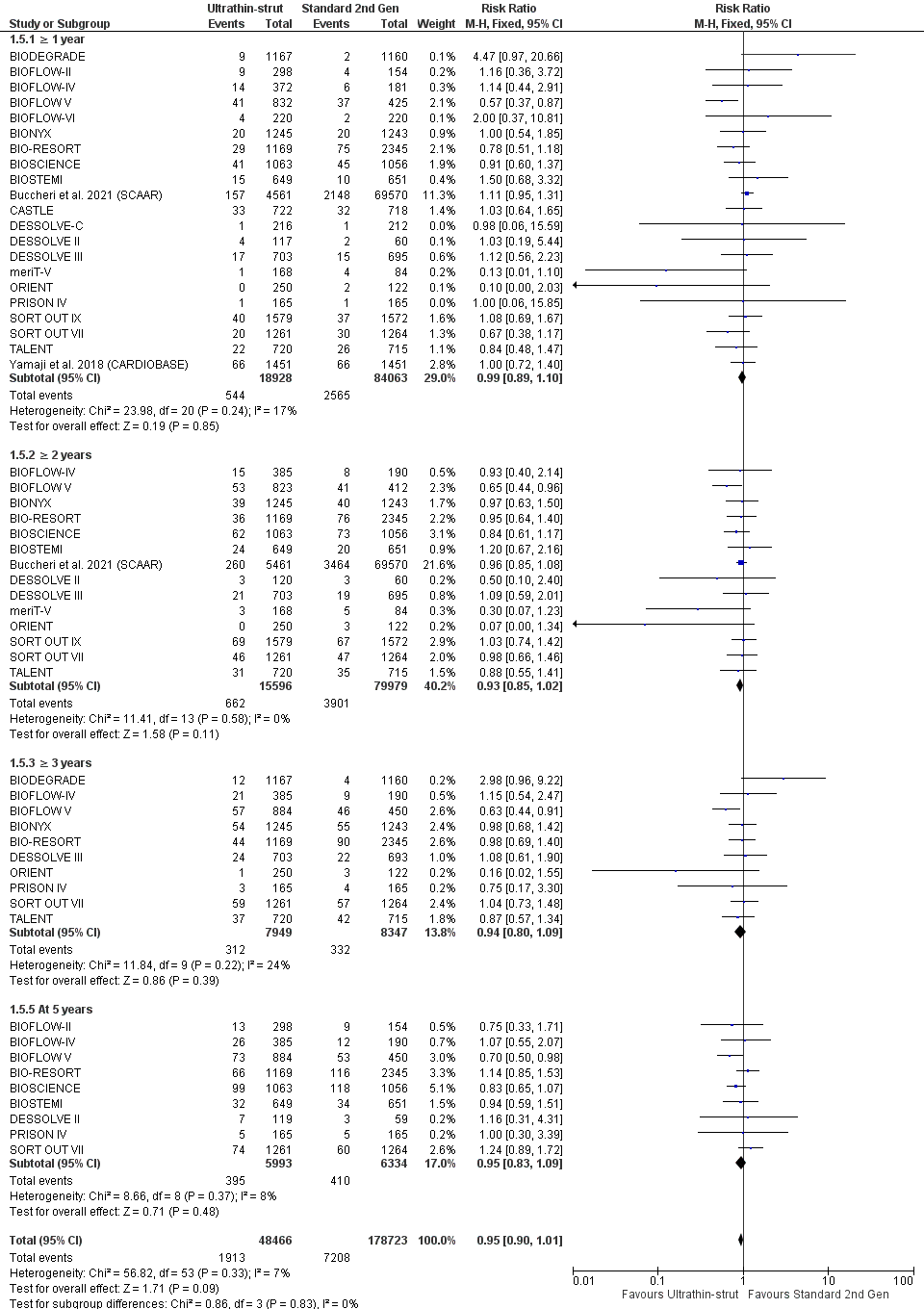


**Figure S18: Forest plot of any myocardial infarction.**


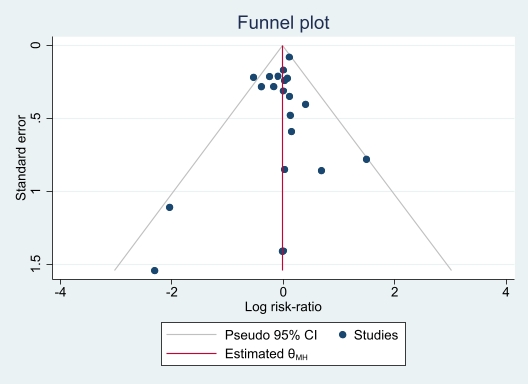


**Figure S19: Funnel plot of any myocardial infarction at ≥ 1 year.**

**
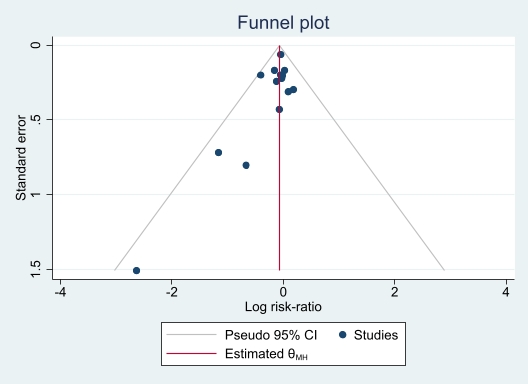
**

**Figure S20: Funnel plot of any myocardial infarction at ≥ 2 years.**


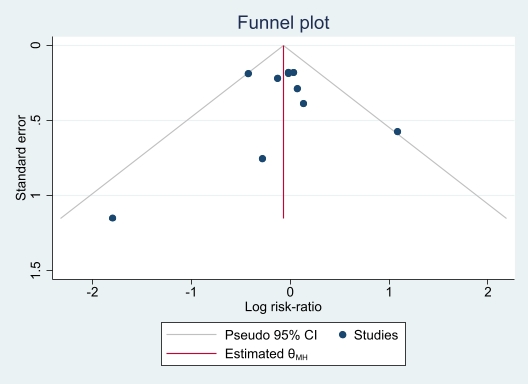


**Figure S21: Funnel plot of any myocardial infarction at ≥ 3 years.**


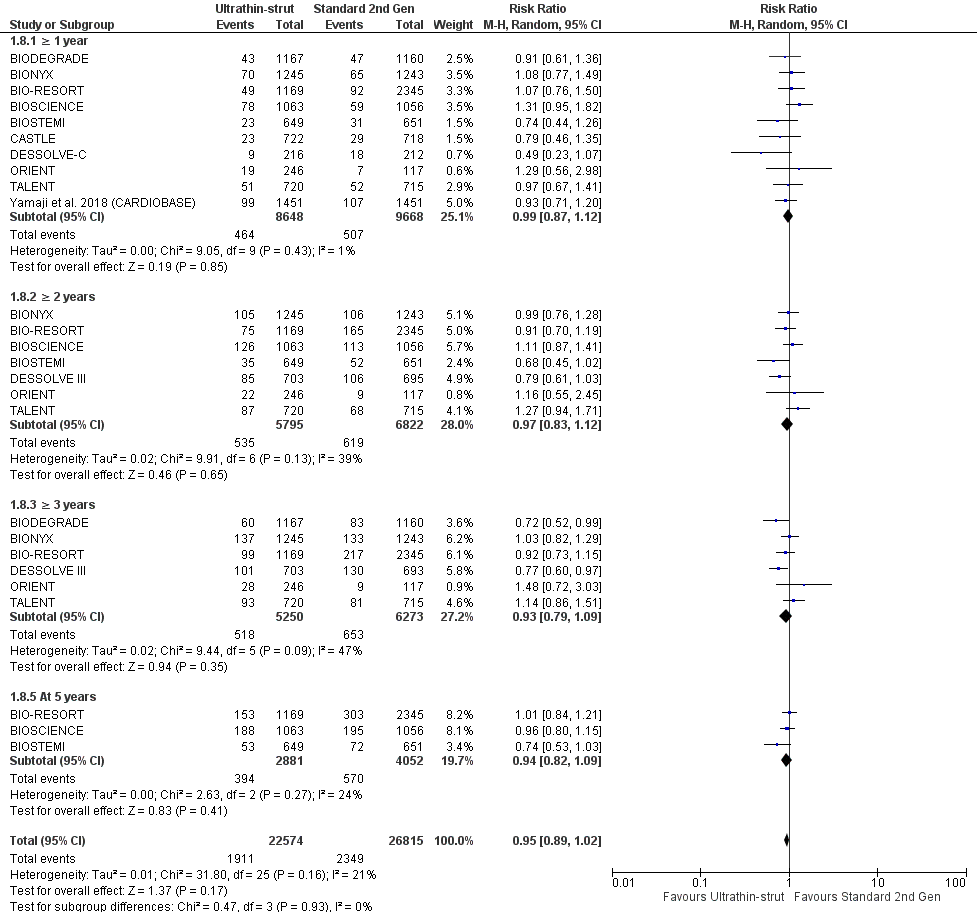


**Figure S22: Forest plot of repeat revascularization at ≥ 1 year.**


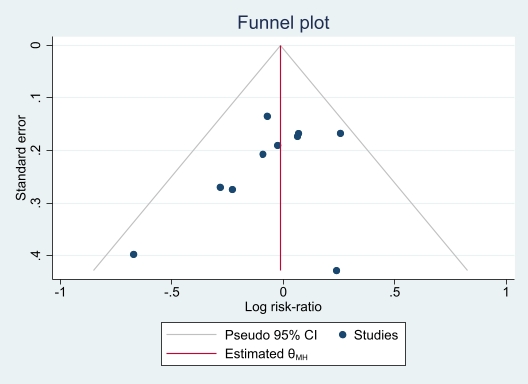


**Figure S23: Funnel plot of any repeat revascularization at ≥ 1 years.**


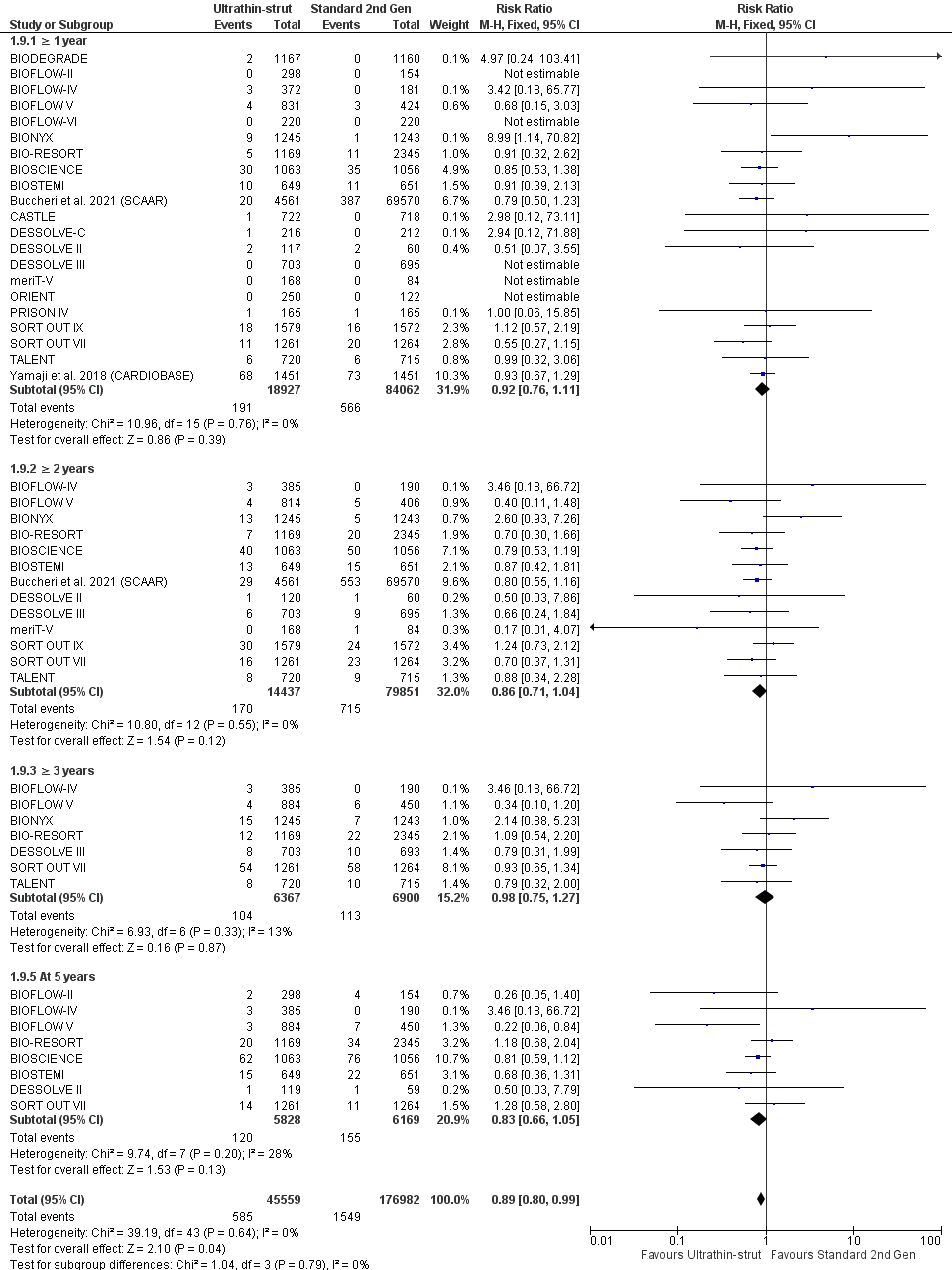


**Figure S24: Forest plot of any definite or probable stent thrombosis.**


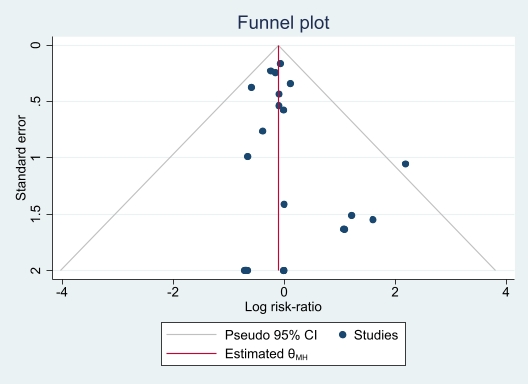


**Figure S25: Funnel plot of any definite or probable stent thrombosis at ≥ 1 year**


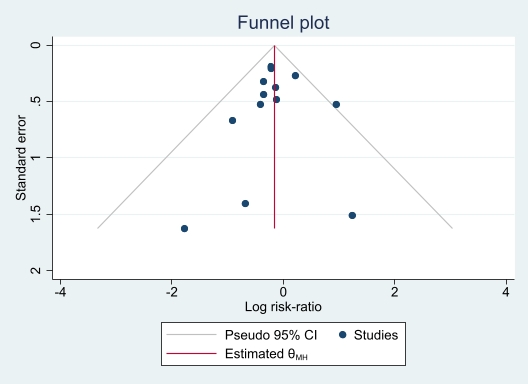


**Figure S26: Funnel plot of any definite or probable stent thrombosis at ≥ 2 years.**


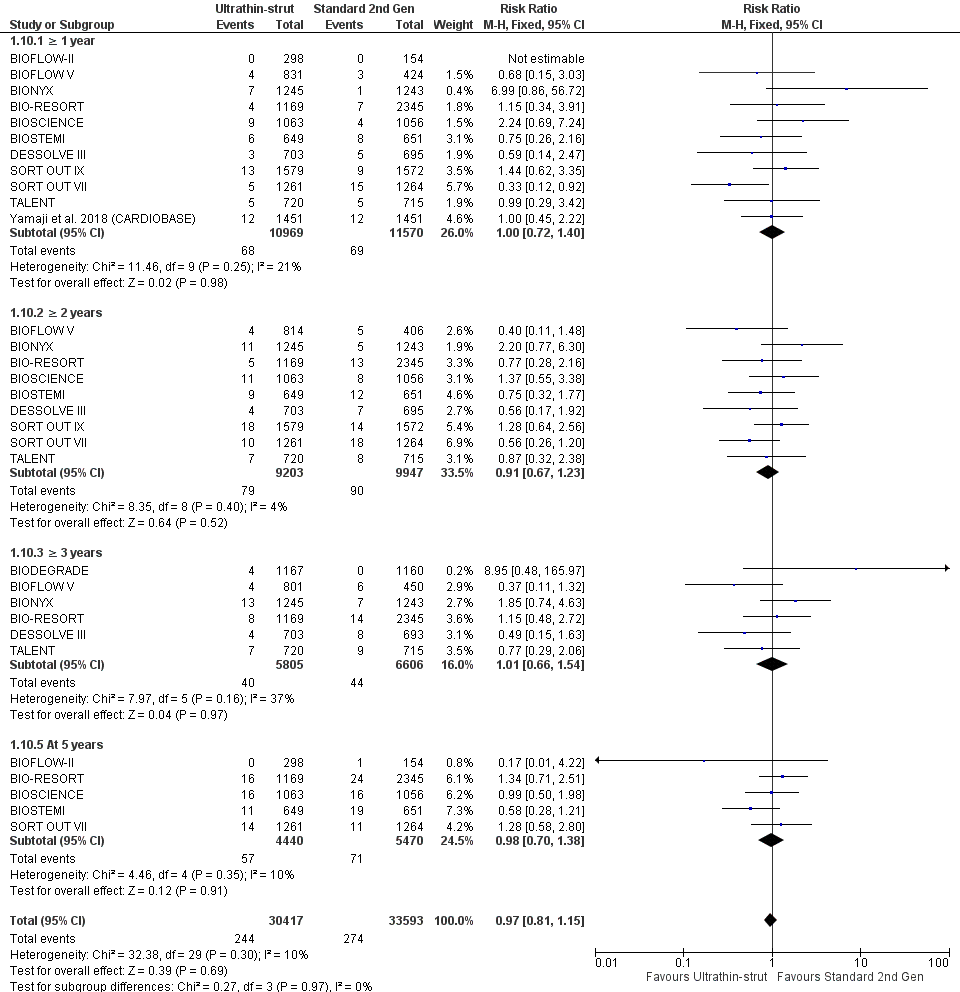


**Figure S27: Forest plot of definite stent thrombosis.**


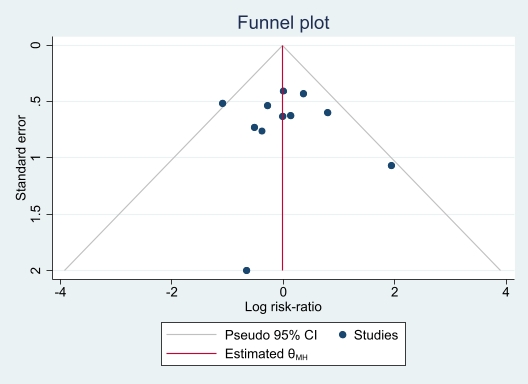


**Figure S28: Funnel plot of definite stent thrombosis at ≥ 1 year.**


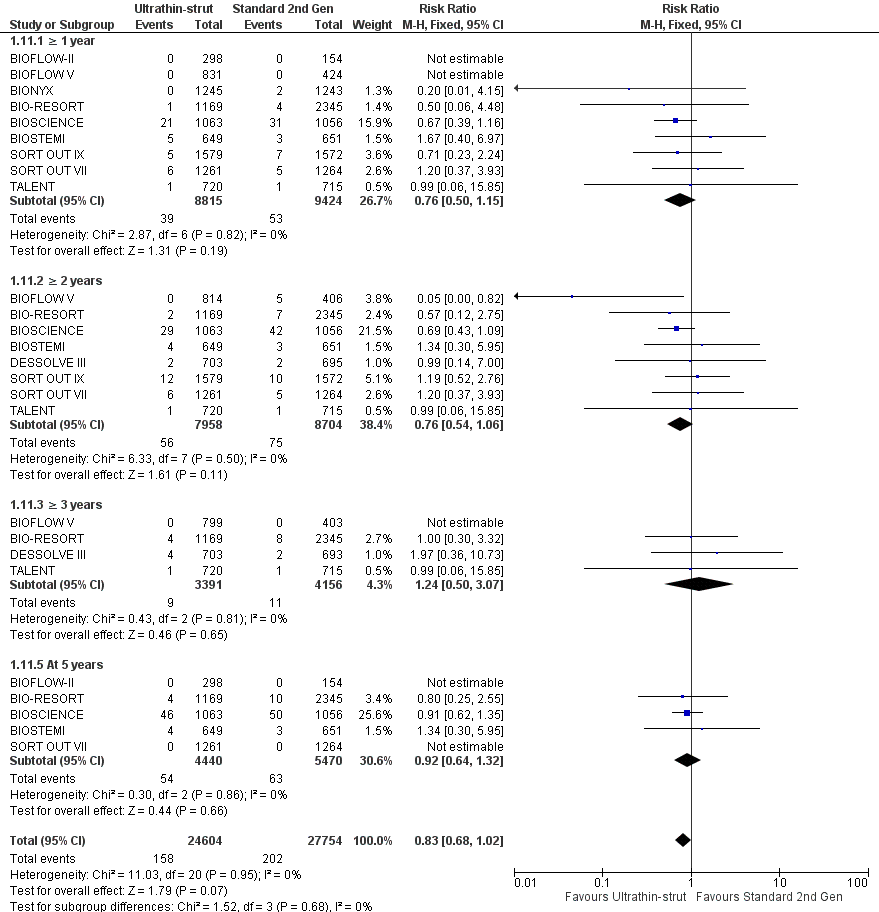


**Figure S29: Forest plot of probable stent thrombosis.**


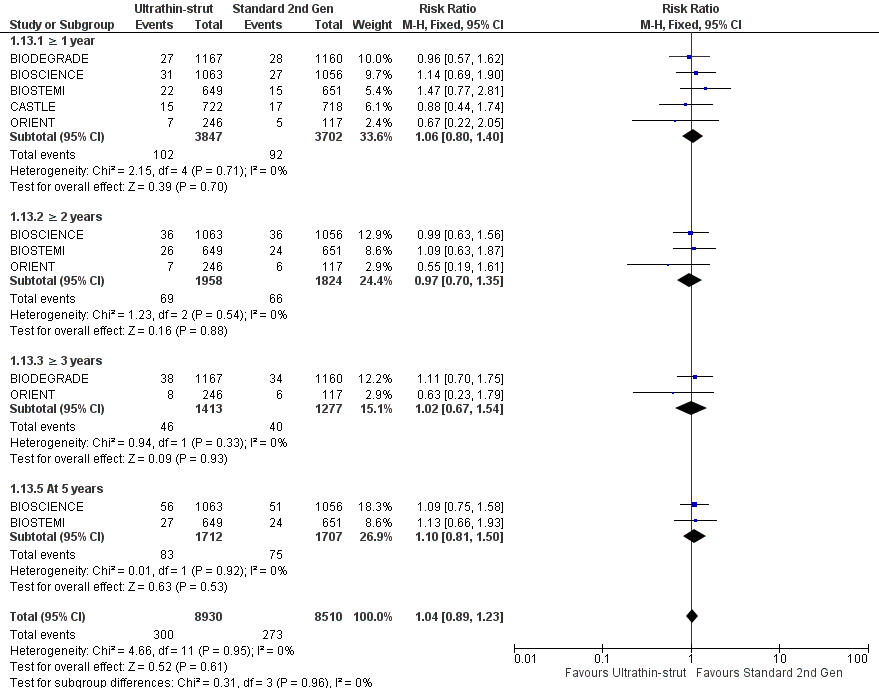


**Figure S30: Forest plot of bleeding.**


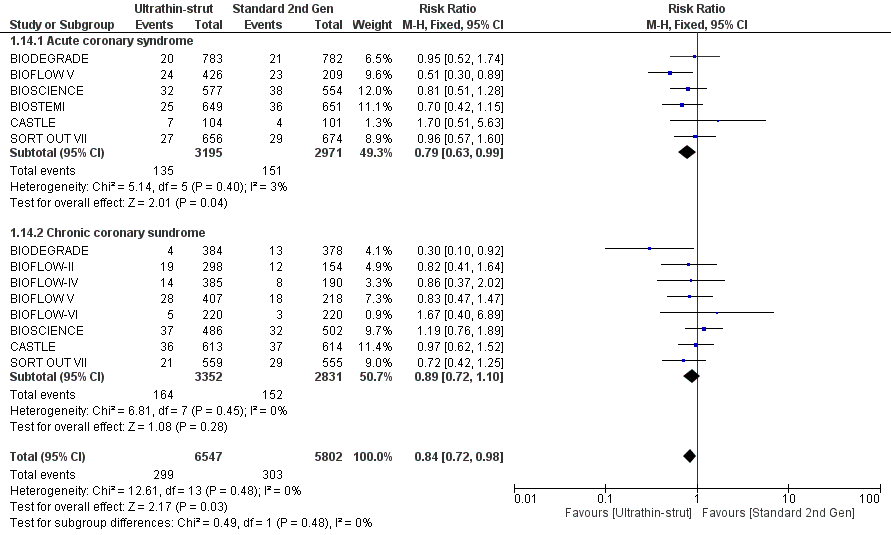


Figure S31A. TLF subgroup analysis at 1 year


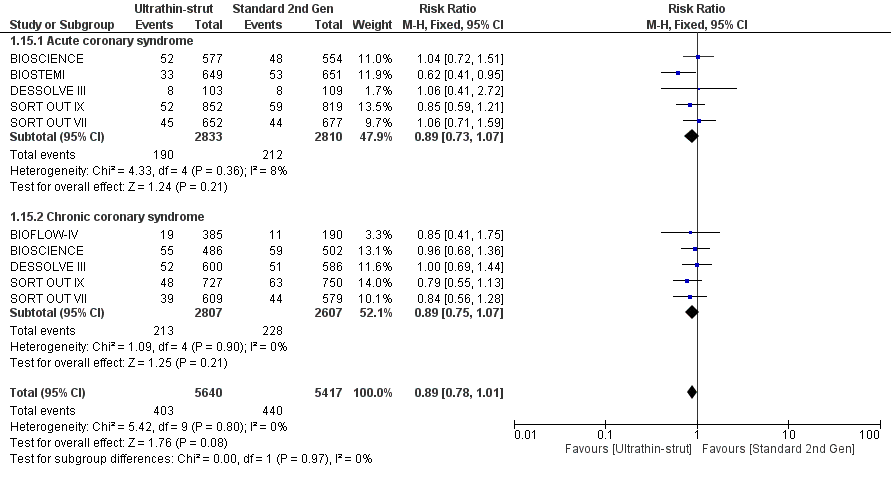


Figure S31B. TLF subgroup analysis at 2 year


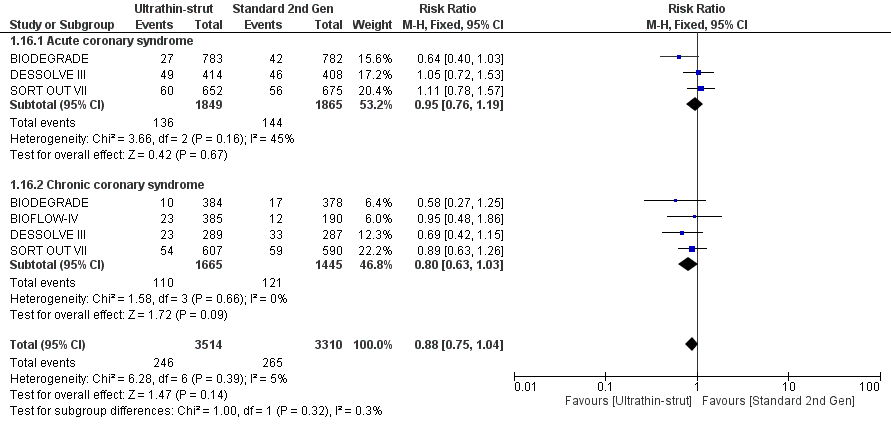


Figure S31C. TLF subgroup analysis at 3 year


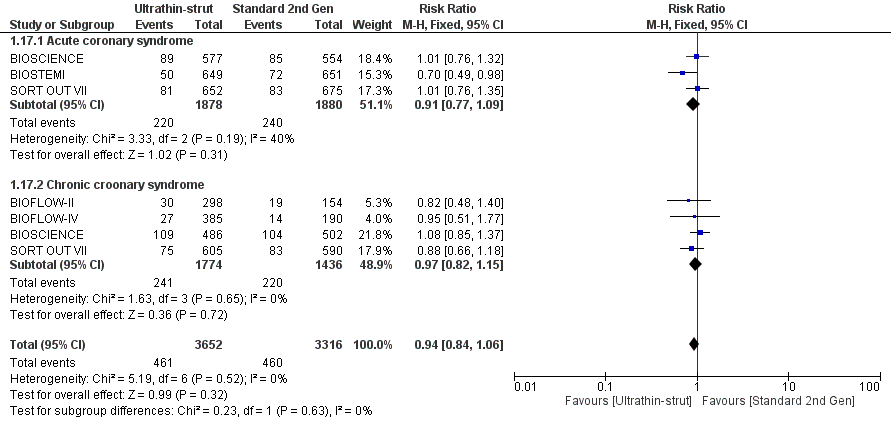


Figure S31D. TLF subgroup analysis at 5 year
